# Supplementary figures and images for: Genome-wide analysis clarifies the population genetic structure of wild gilthead sea bream (Sparus aurata)
Source: PLoS One. 2021 Jan 11;16(1):e0236230. doi: 10.1371/journal.pone.0236230 (PMC7799848; doi:10.1371/journal.pone.0236230)

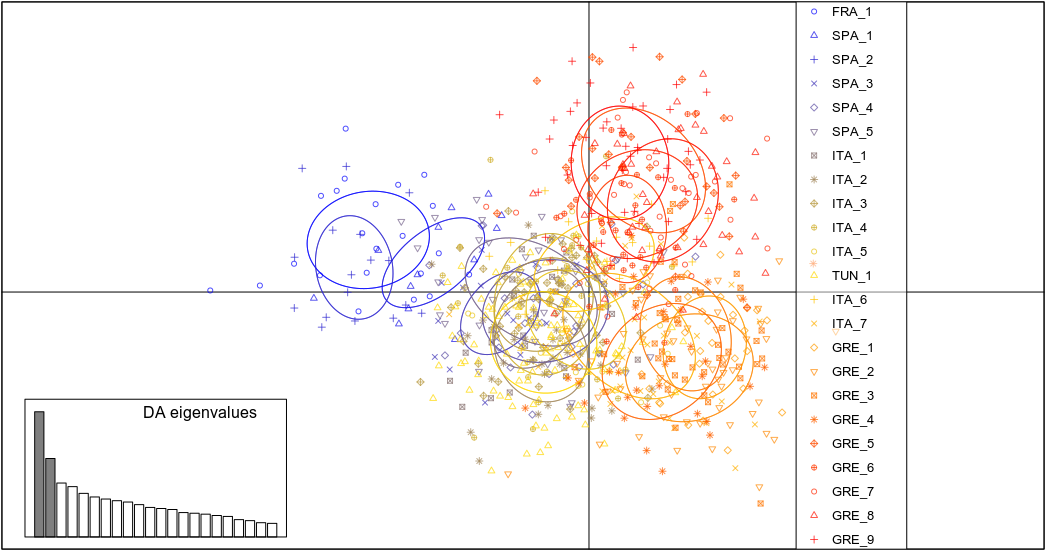

Supplement: S1 Fig — The barchart indicates the Discriminant Axes eigenvalue. Circles represent 95% inertia ellipses. (PNG) [file pone.0236230.s001.png]

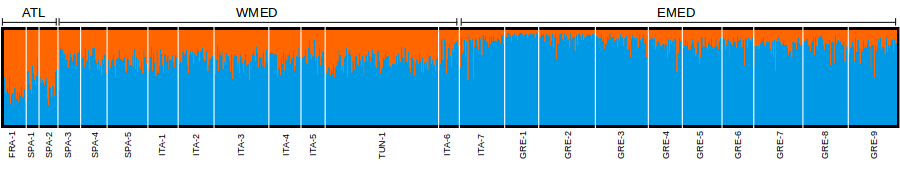

Supplement: S3 Fig — (TIF) [file pone.0236230.s003.tif]

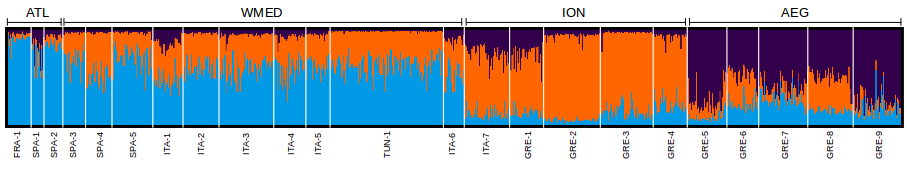

Supplement: S4 Fig — (TIF) [file pone.0236230.s004.tif]
