# Supplementary material for: Genome-wide analysis clarifies the population genetic structure of wild gilthead sea bream (Sparus aurata)
Source: PLoS One. 2021 Jan 11;16(1):e0236230. doi: 10.1371/journal.pone.0236230 (PMC7799848; doi:10.1371/journal.pone.0236230)
Supplement: S2 Fig — Populations are ordered according to the four genetic clusters identified. For markers identified by Bayenv, values of the correlated environmental parameter are indicated by blue bars. (PDF) [file pone.0236230.s002.pdf]

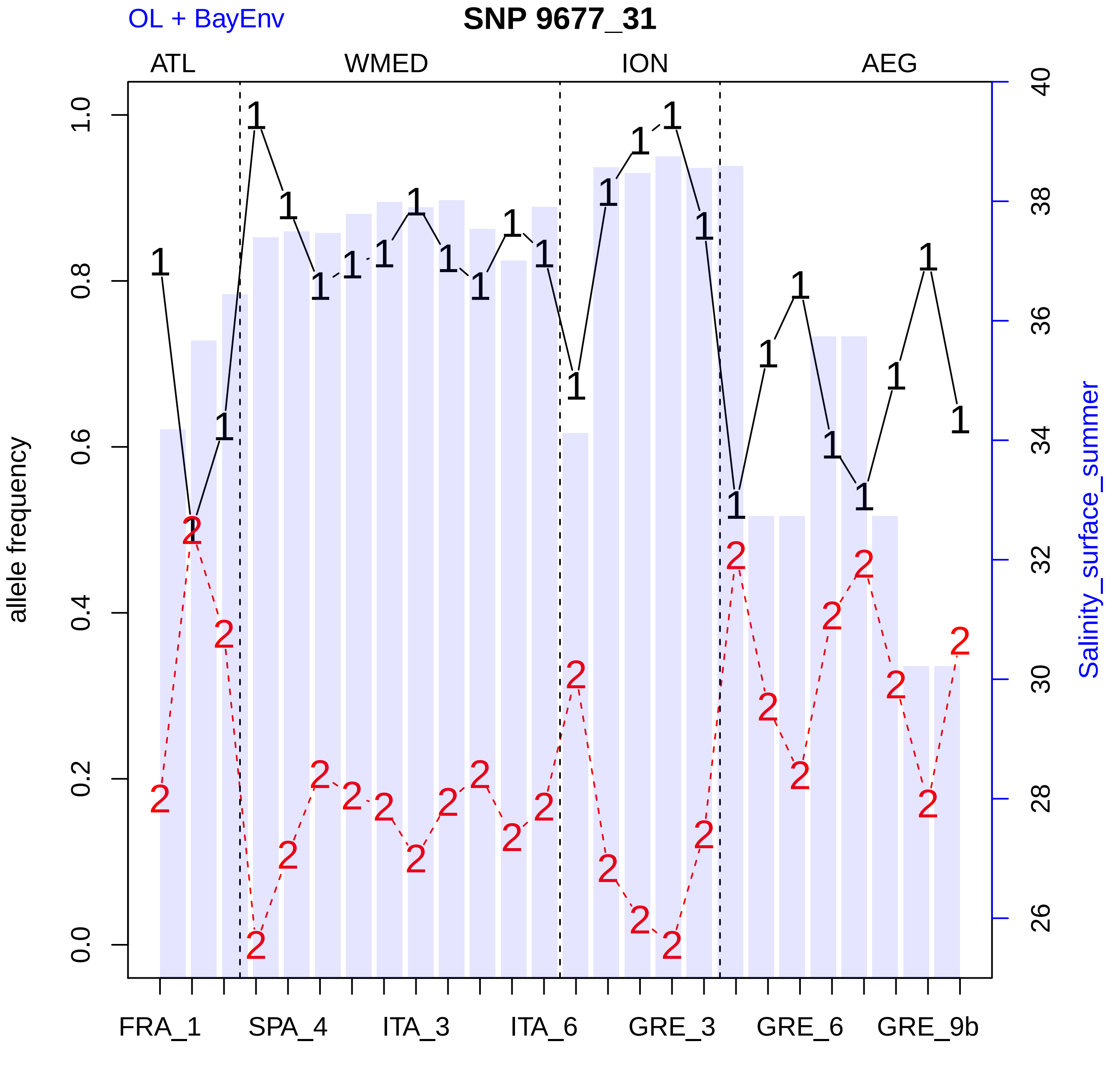

OL + BayEnv
SNP 9677_31
ATL
WMED
ION
AEG
allele frequency
Salinity_surface_summer
0.0
0.2
0.4
0.6
0.8
1.0
26
28
30
32
34
36
38
40
FRA_1
SPA_4
ITA_3
ITA_6
GRE_3
GRE_6
GRE_9b

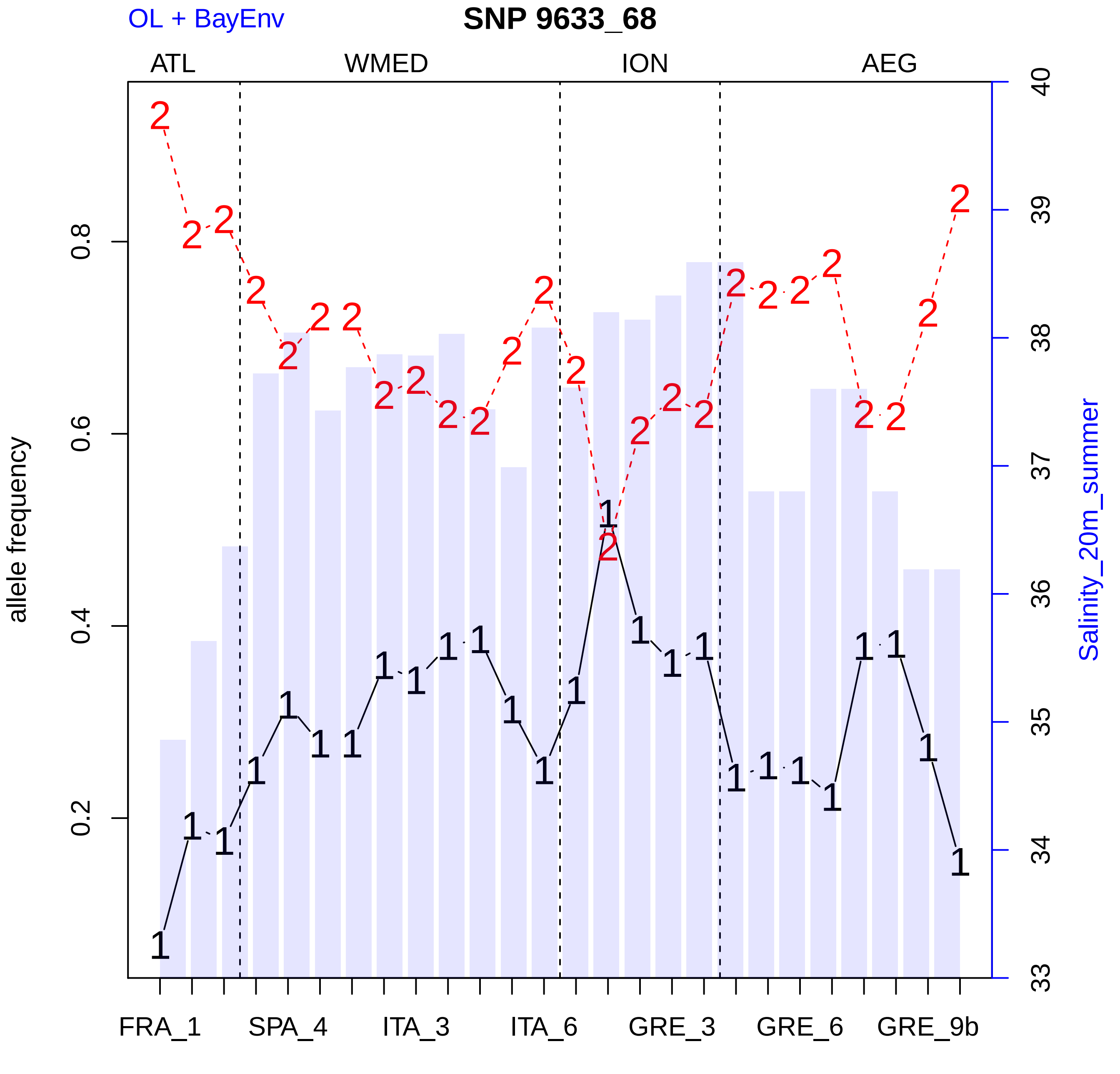

OL + BayEnv
SNP 9633_68
ATL
WMED
ION
AEG
allele frequency
0.2
0.4
0.6
0.8
Salinity_20m_summer
33
34
35
36
37
38
39
40
FRA_1
SPA_4
ITA_3
ITA_6
GRE_3
GRE_6
GRE_9b

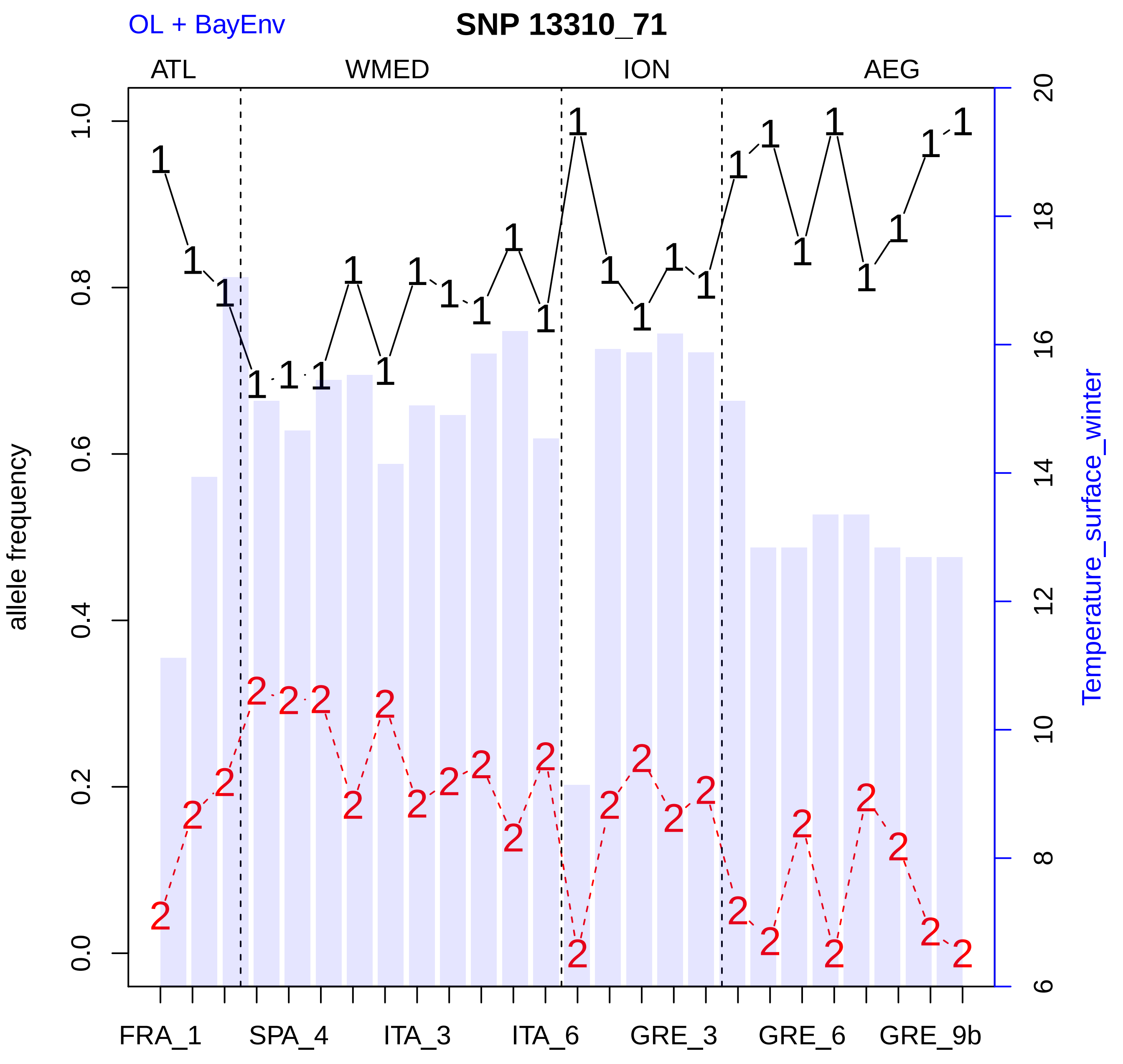

OL + BayEnv
SNP 13310_71
ATL
WMED
ION
AEG
allele frequency
Temperature_surface_winter
0.0
0.2
0.4
0.6
0.8
1.0
6
8
10
12
14
16
18
20
FRA_1
SPA_4
ITA_3
ITA_6
GRE_3
GRE_6
GRE_9b

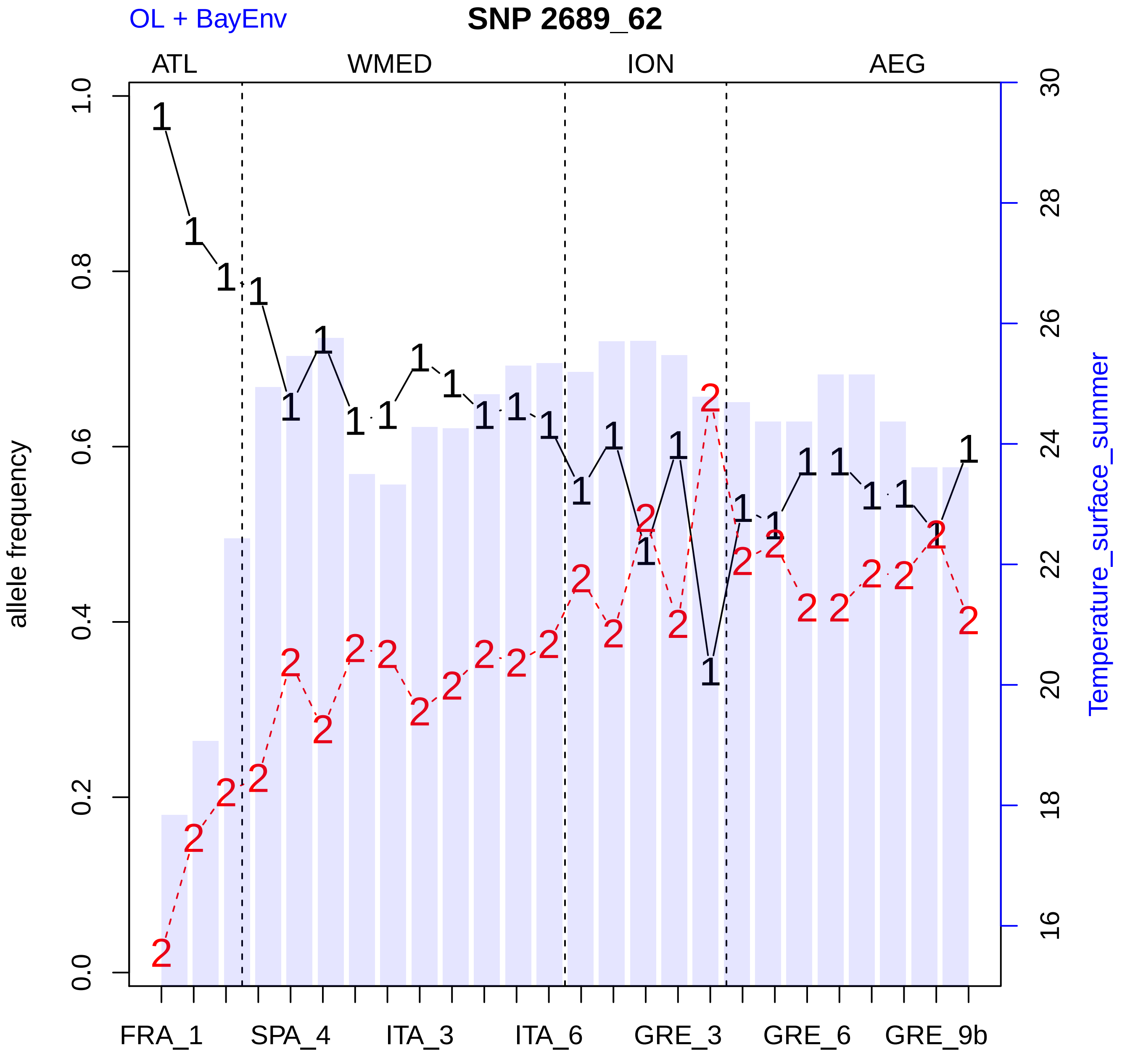

OL + BayEnv
SNP 2689_62
ATL
WMED
ION
AEG
allele frequency
Temperature_surface_summer
0.0
0.2
0.4
0.6
0.8
1.0
16
18
20
22
24
26
28
30
FRA_1
SPA_4
ITA_3
ITA_6
GRE_3
GRE_6
GRE_9b

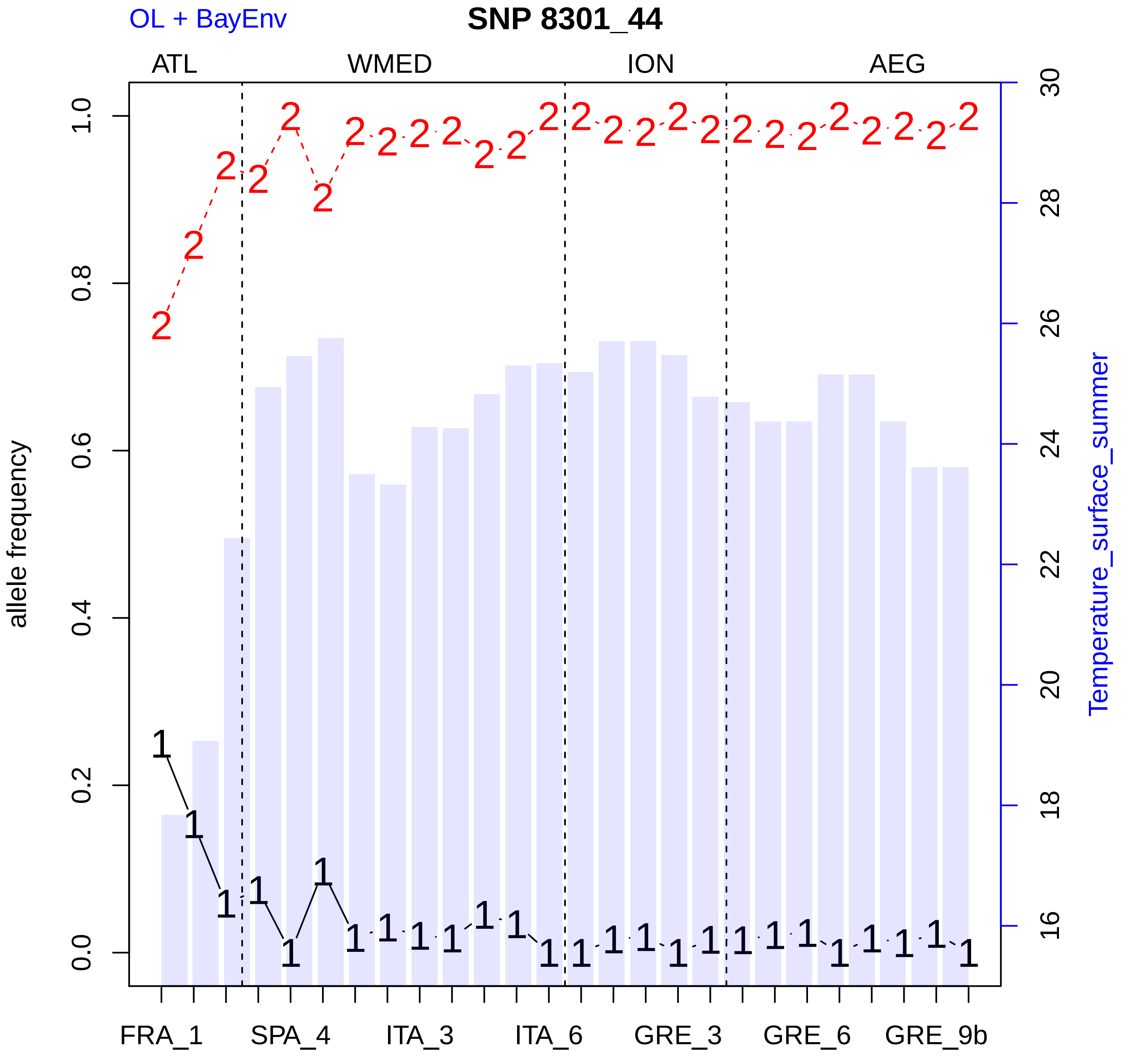

OL + BayEnv
SNP 8301_44
ATL
WMED
ION
AEG
allele frequency
0.0
0.2
0.4
0.6
0.8
1.0
Temperature_surface_summer
16
18
20
22
24
26
28
30
FRA_1
SPA_4
ITA_3
ITA_6
GRE_3
GRE_6
GRE_9b

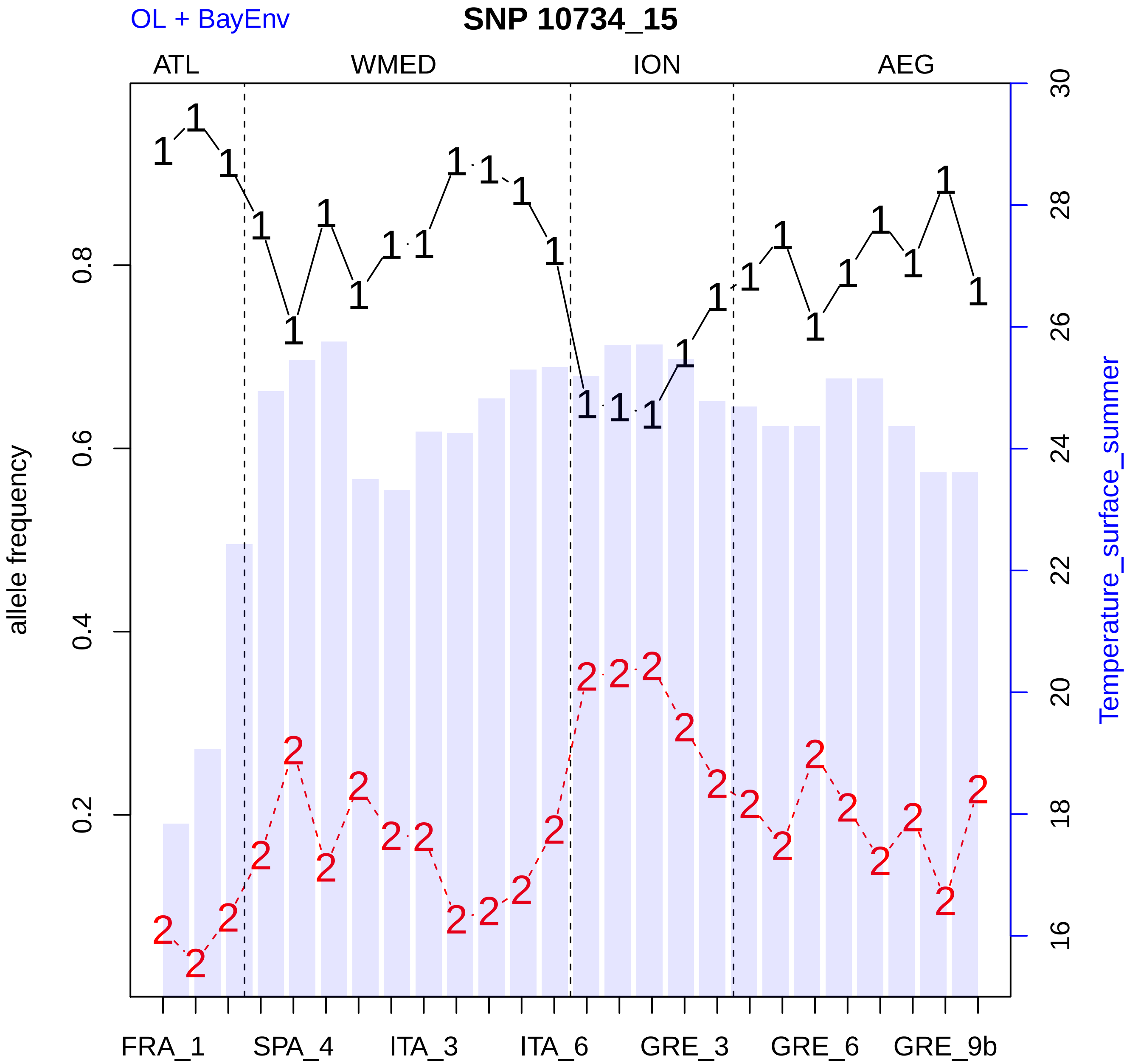
OL + BayEnv
SNP 10734_15
ATL
WMED
ION
AEG
allele frequency
0.2
0.4
0.6
0.8
Temperature_surface_summer
16
18
20
22
24
26
28
30
FRA_1
SPA_4
ITA_3
ITA_6
GRE_3
GRE_6
GRE_9b

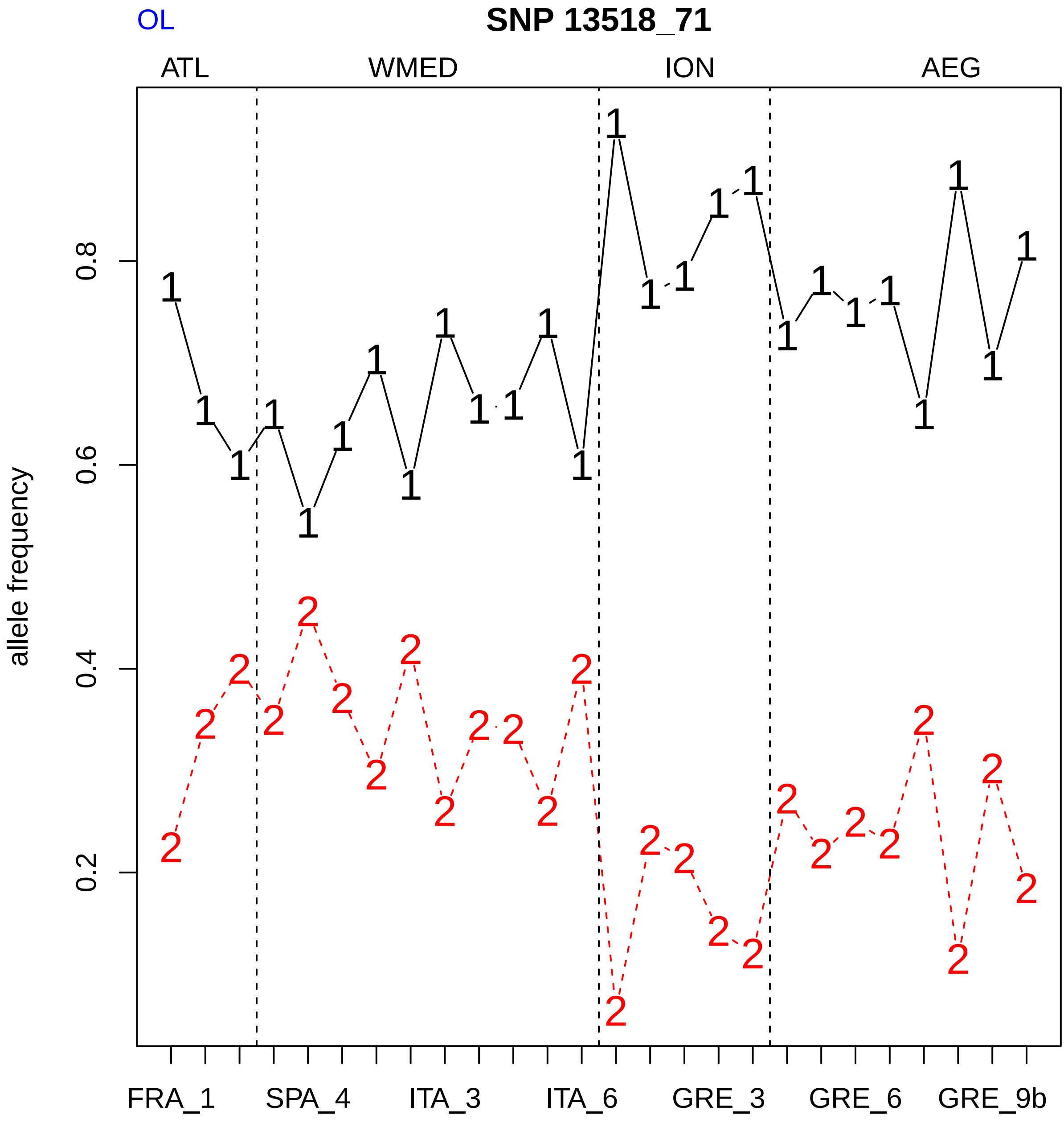
OL
SNP 13518_71
ATL
WMED
ION
AEG
allele frequency
0.2
0.4
0.6
0.8
FRA_1
SPA_4
ITA_3
ITA_6
GRE_3
GRE_6
GRE_9b

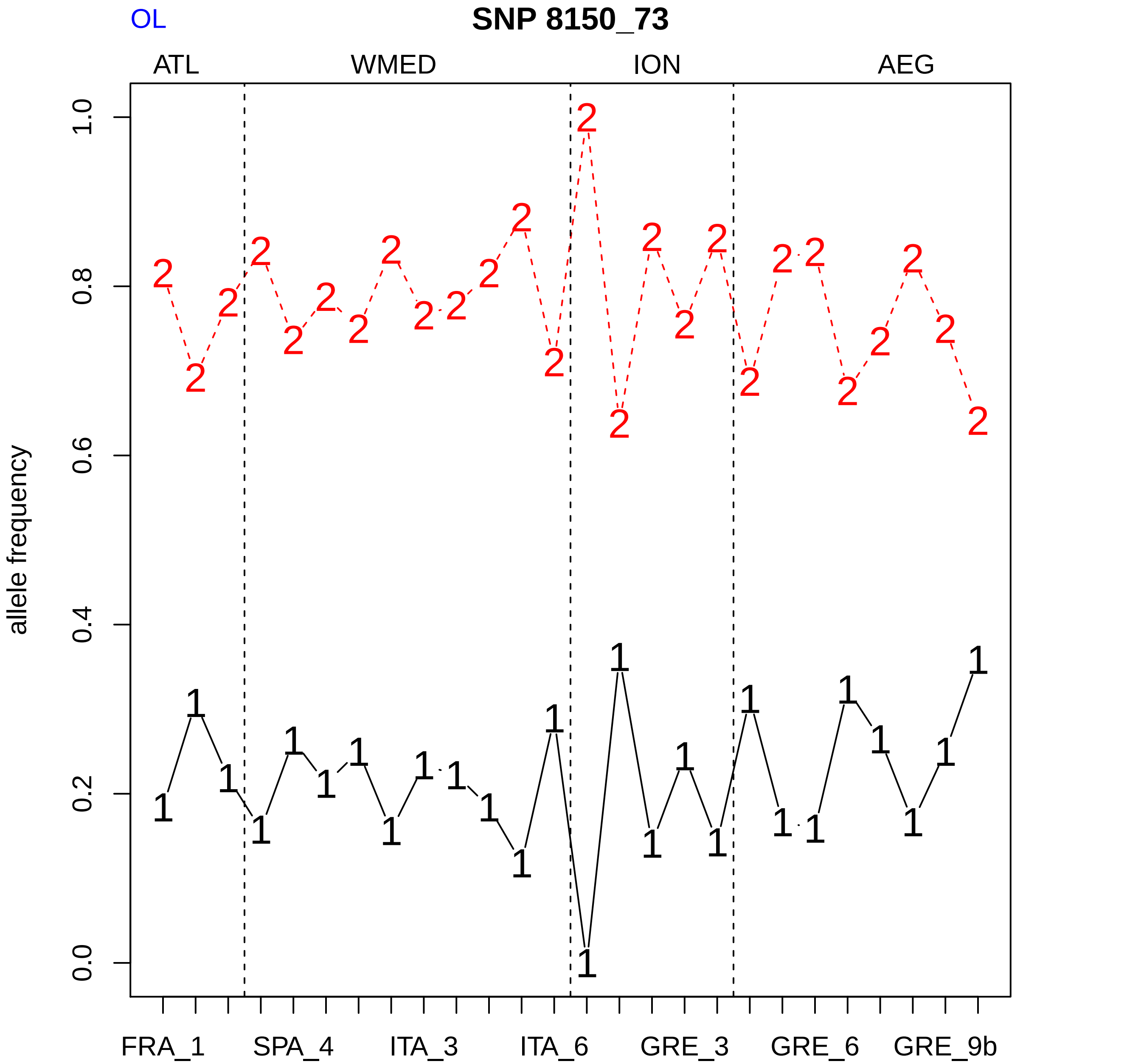
OL
SNP 8150_73
ATL
WMED
ION
AEG
allele frequency
0.0
0.2
0.4
0.6
0.8
1.0
FRA_1
SPA_4
ITA_3
ITA_6
GRE_3
GRE_6
GRE_9b

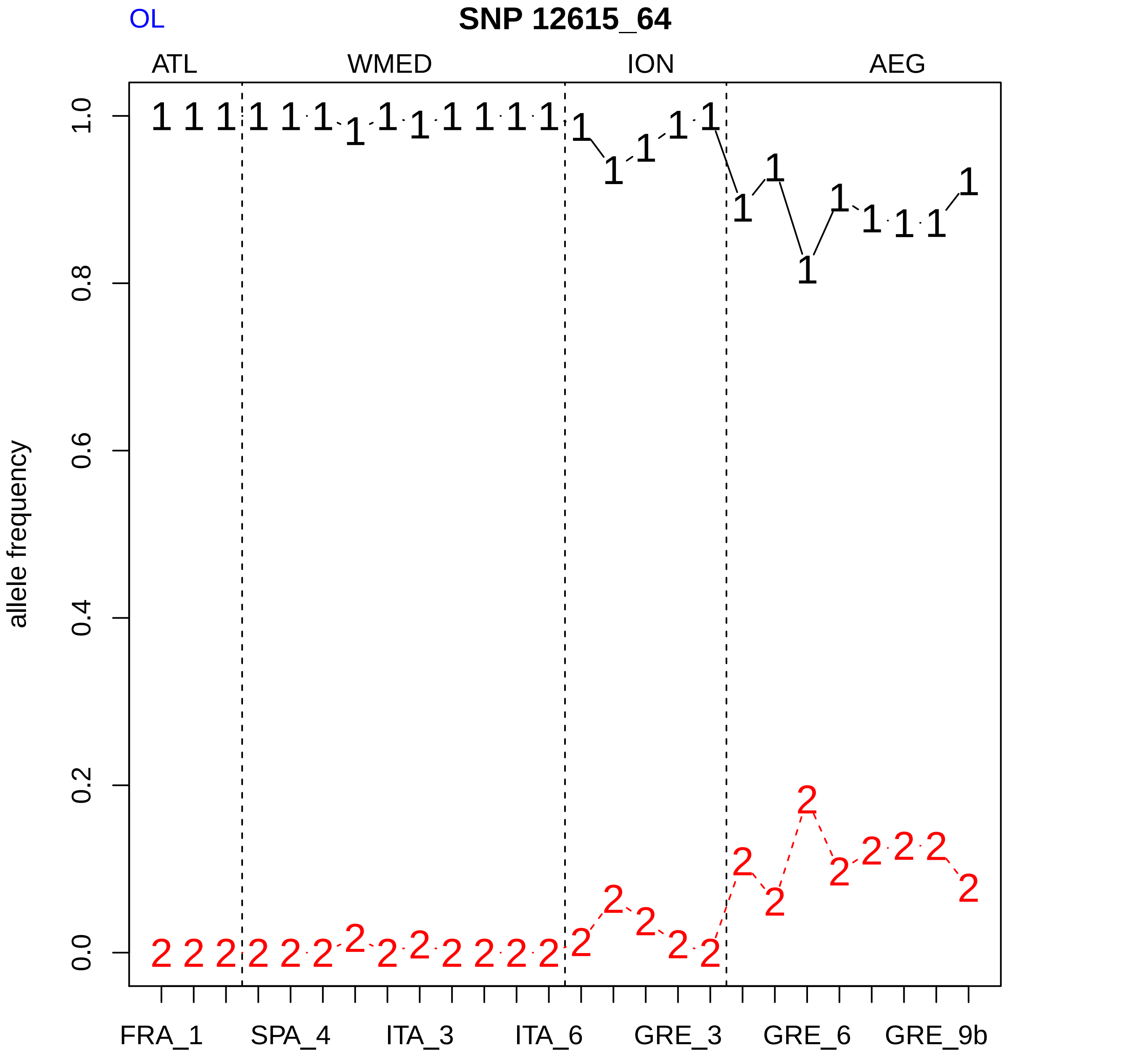
OL
SNP 12615_64
ATL
WMED
ION
AEG
allele frequency
0.0
0.2
0.4
0.6
0.8
1.0
FRA_1
SPA_4
ITA_3
ITA_6
GRE_3
GRE_6
GRE_9b

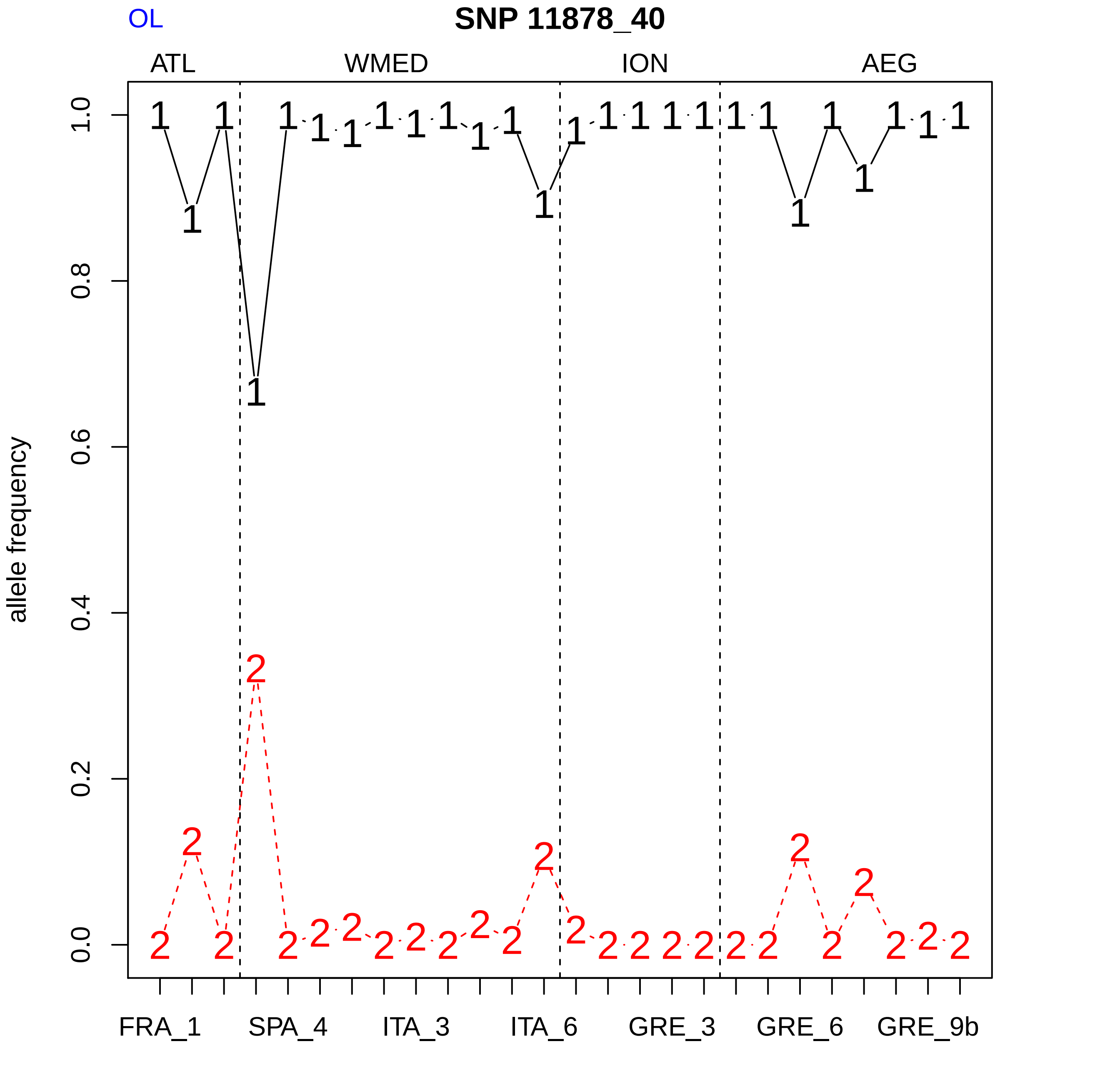

OL
SNP 11878_40
ATL
WMED
ION
AEG
allele frequency
0.0
0.2
0.4
0.6
0.8
1.0
FRA_1
SPA_4
ITA_3
ITA_6
GRE_3
GRE_6
GRE_9b

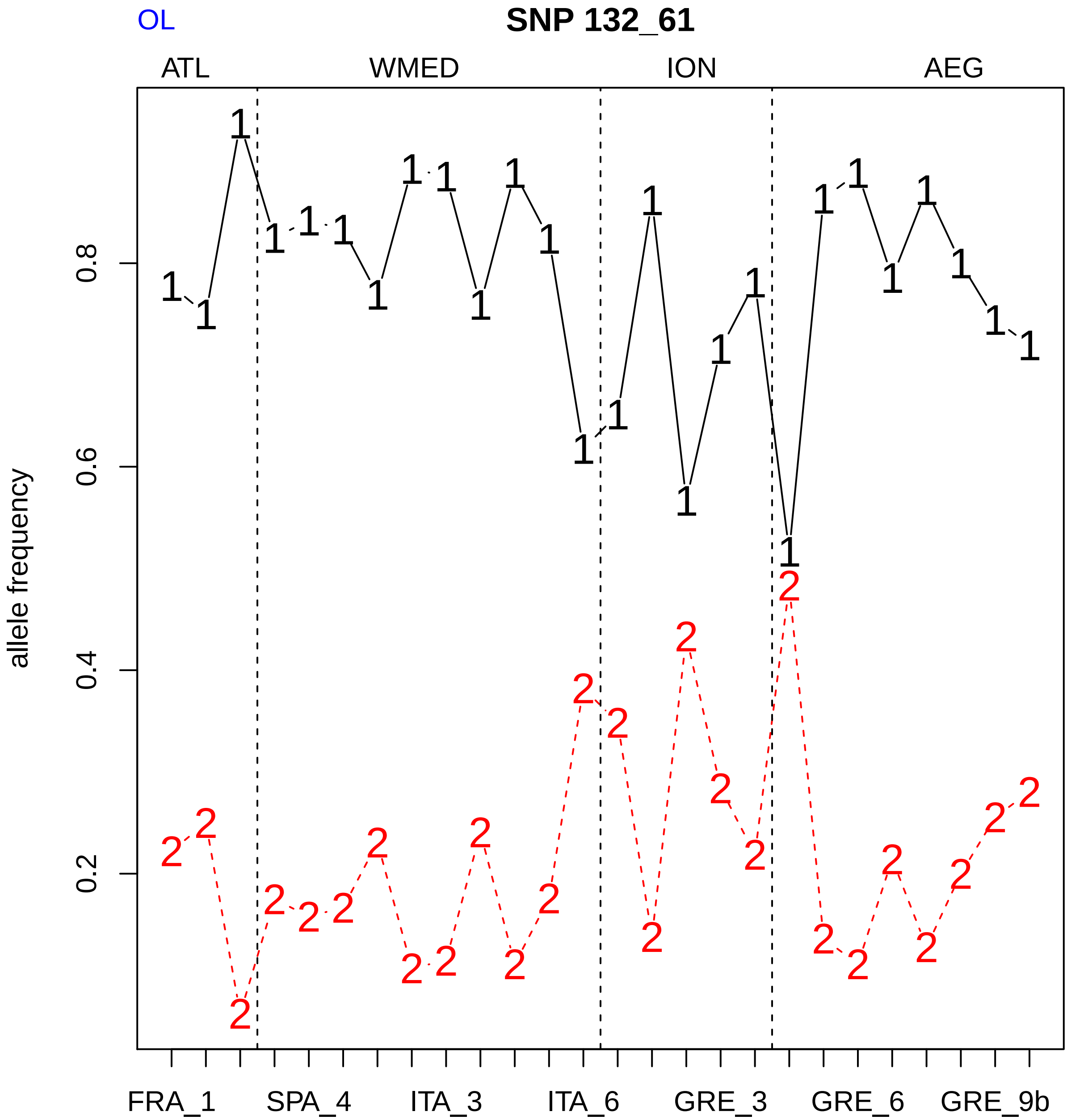
OL
SNP 132_61
ATL
WMED
ION
AEG
allele frequency
0.2
0.4
0.6
0.8
FRA_1
SPA_4
ITA_3
ITA_6
GRE_3
GRE_6
GRE_9b

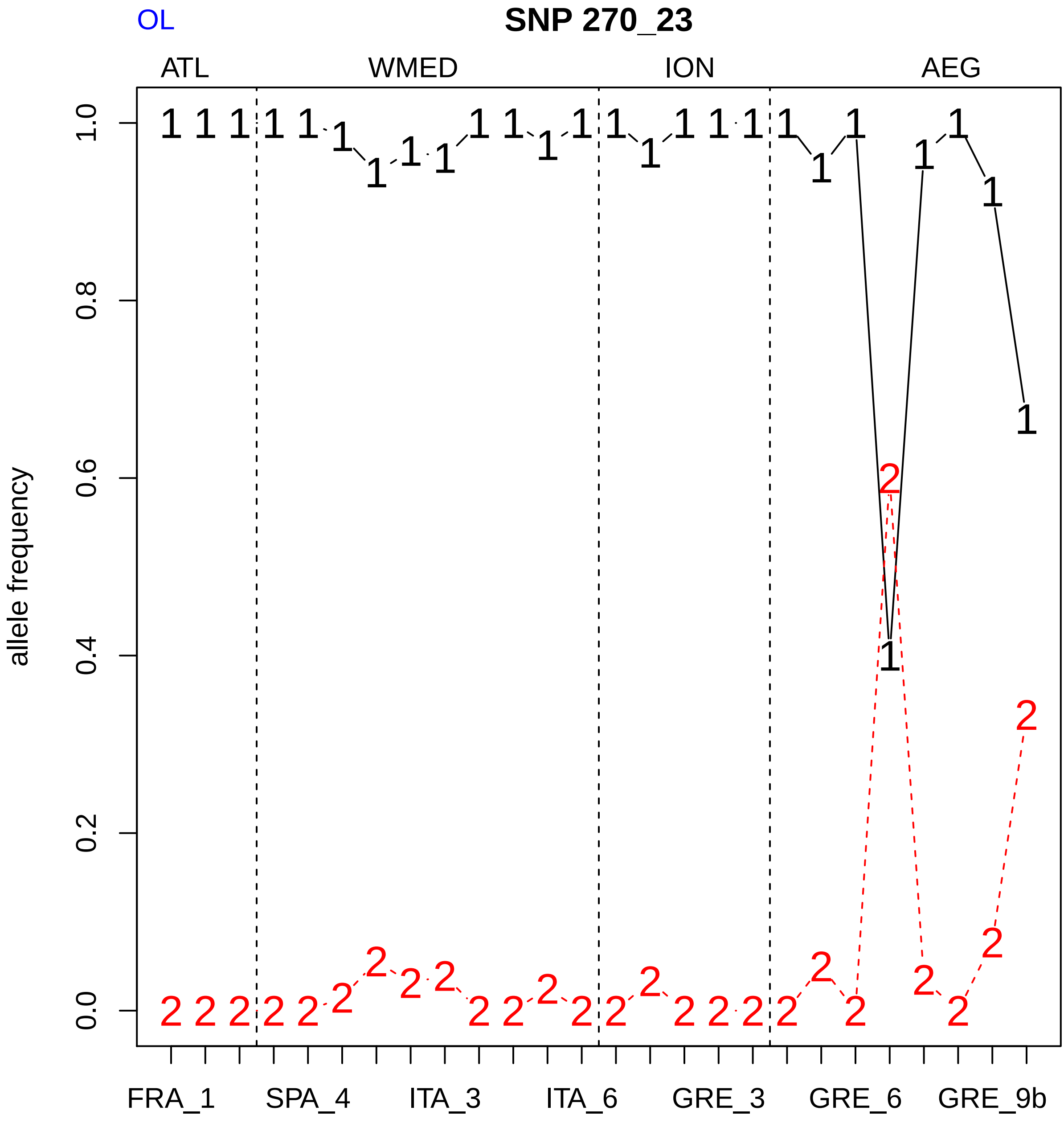

OL
SNP 270_23
ATL
WMED
ION
AEG
allele frequency
0.0
0.2
0.4
0.6
0.8
1.0
FRA_1
SPA_4
ITA_3
ITA_6
GRE_3
GRE_6
GRE_9b

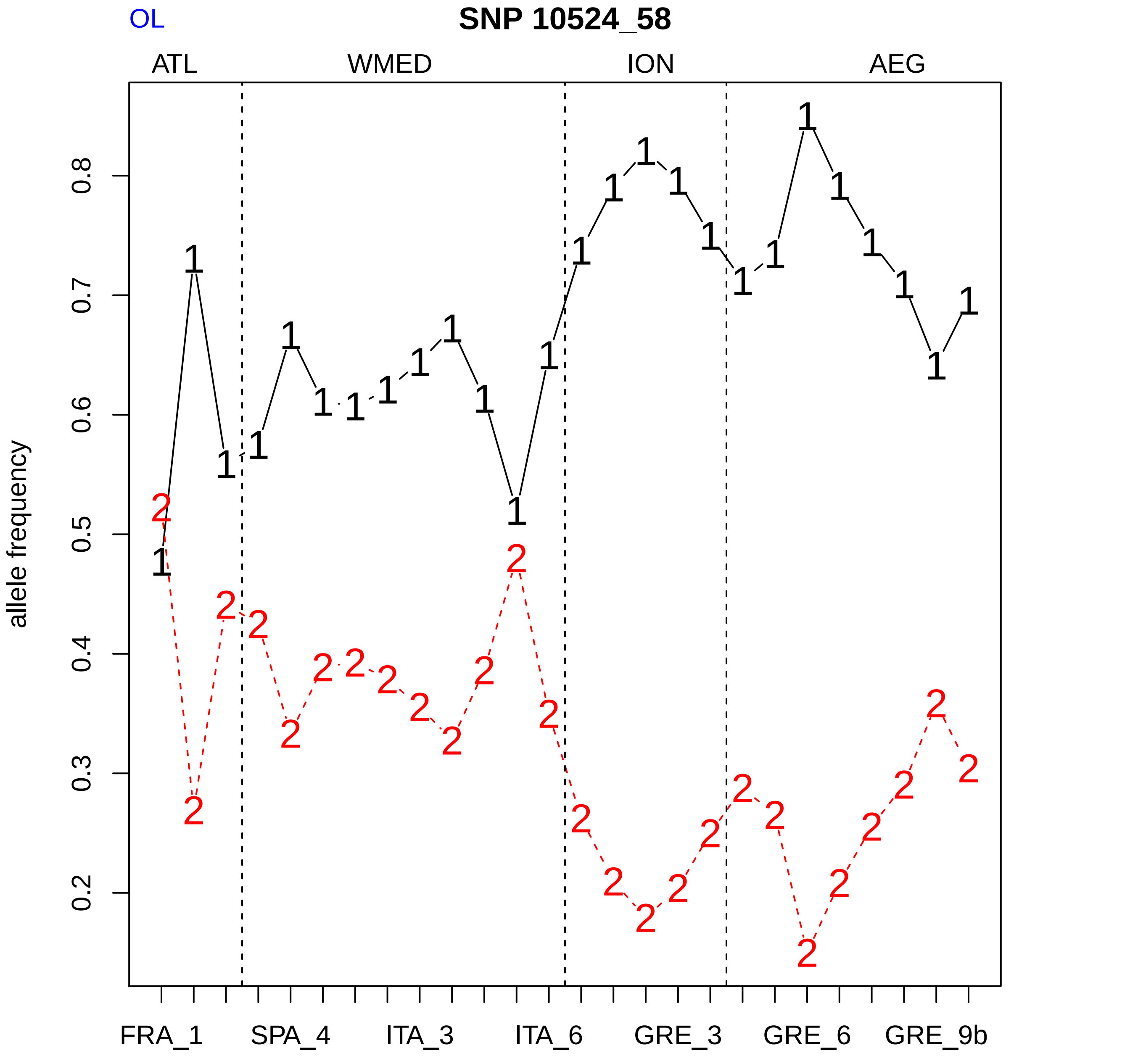

OL
SNP 10524_58
ATL
WMED
ION
AEG
allele frequency
0.2
0.3
0.4
0.5
0.6
0.7
0.8
FRA_1
SPA_4
ITA_3
ITA_6
GRE_3
GRE_6
GRE_9b

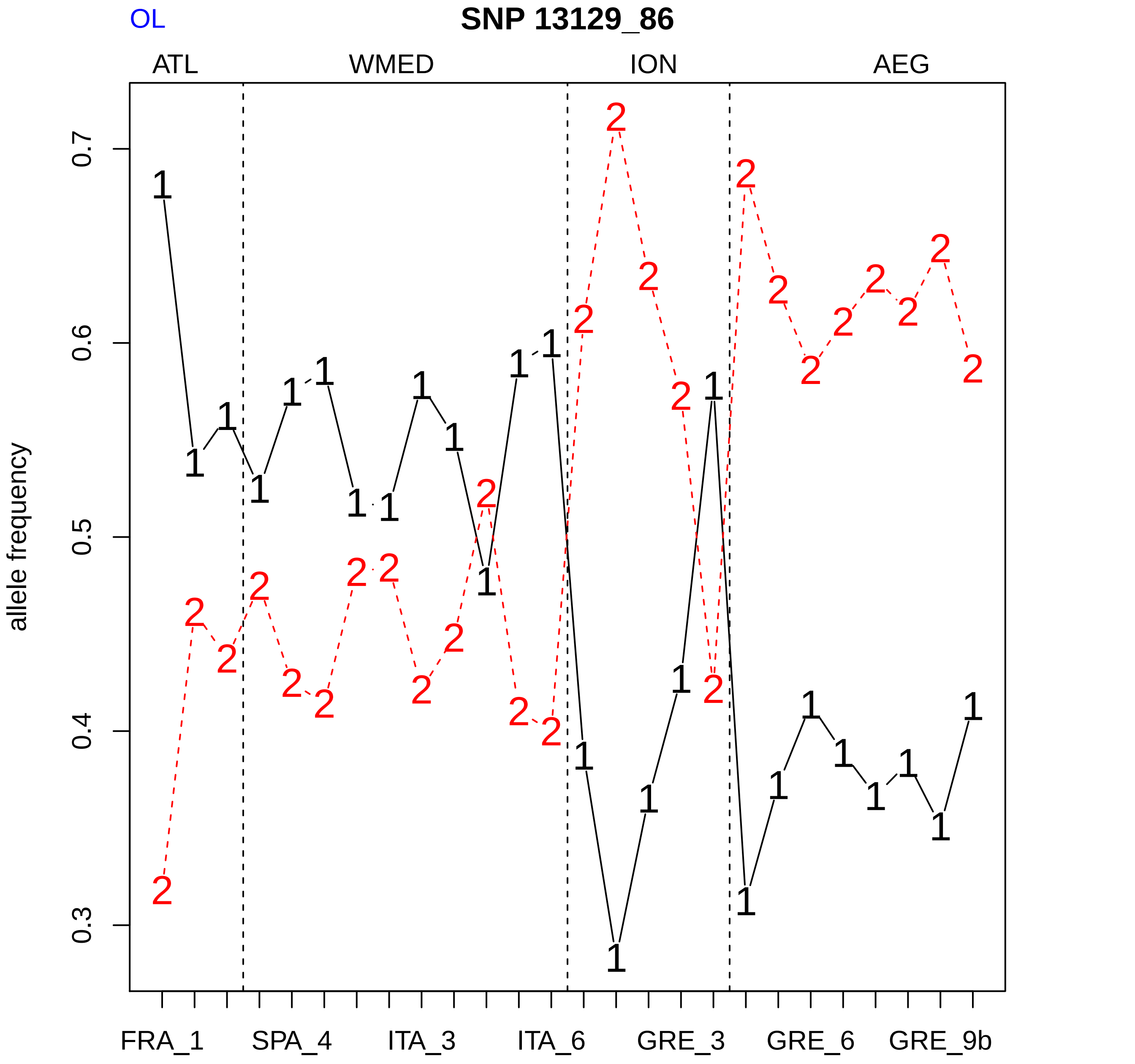
OL
SNP 13129_86
ATL
WMED
ION
AEG
allele frequency
0.3
0.4
0.5
0.6
0.7
FRA_1
SPA_4
ITA_3
ITA_6
GRE_3
GRE_6
GRE_9b

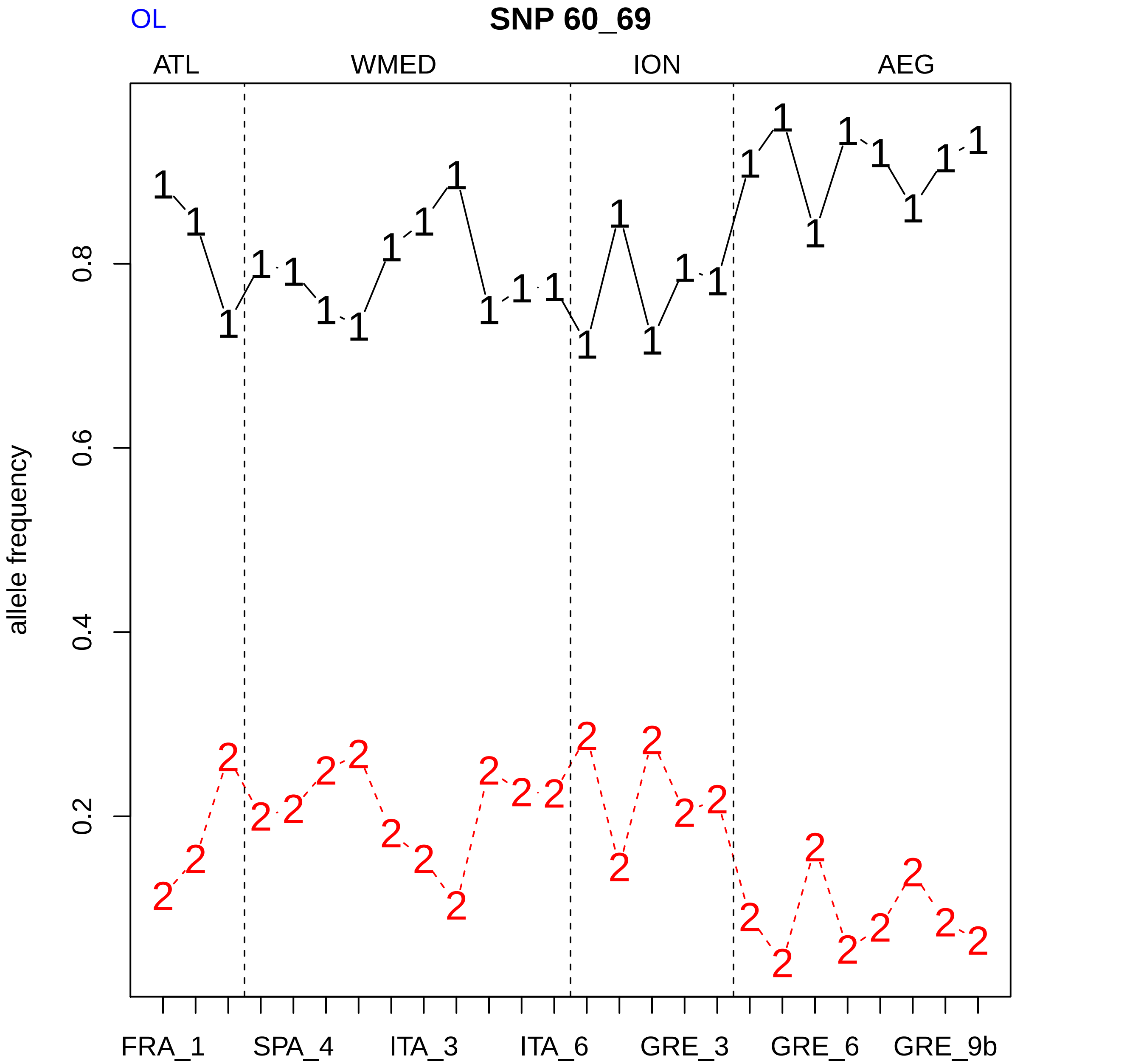

OL
SNP 60_69
ATL
WMED
ION
AEG
allele frequency
0.2
0.4
0.6
0.8
FRA_1
SPA_4
ITA_3
ITA_6
GRE_3
GRE_6
GRE_9b

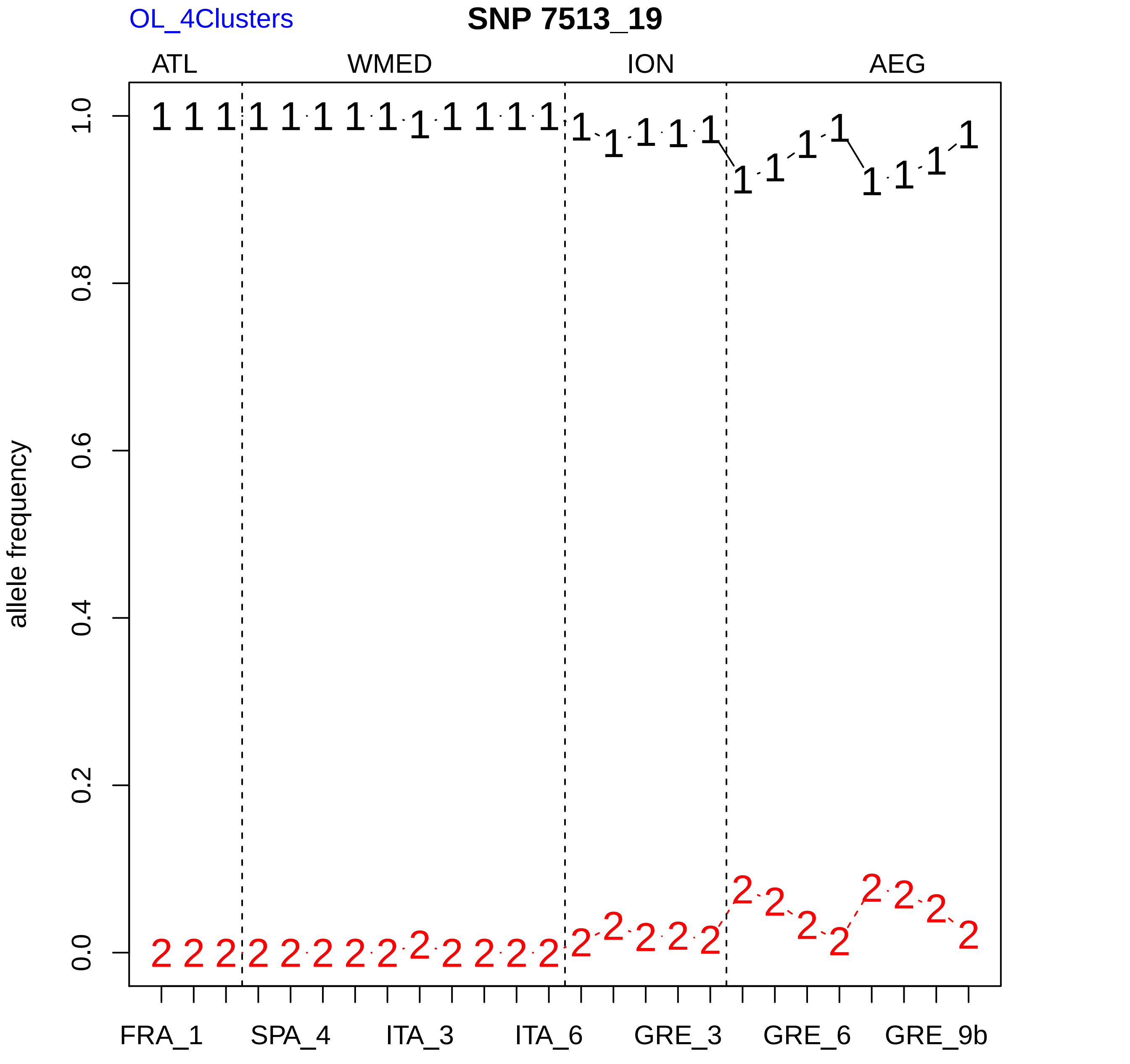
OL_4Clusters
SNP 7513_19
ATL
WMED
ION
AEG
allele frequency
0.0
0.2
0.4
0.6
0.8
1.0
FRA_1
SPA_4
ITA_3
ITA_6
GRE_3
GRE_6
GRE_9b

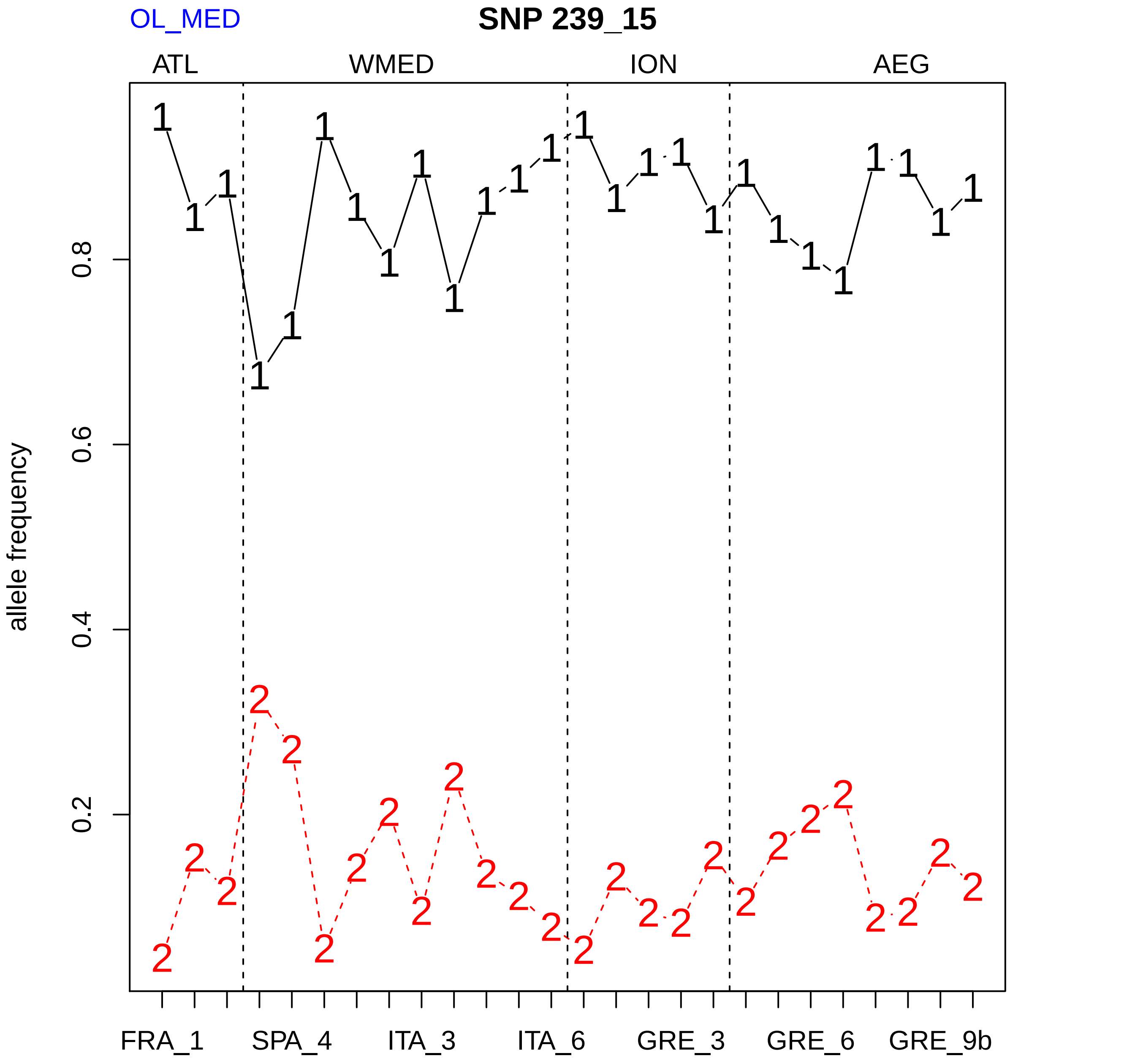

OL_MED
SNP 239_15
ATL
WMED
ION
AEG
allele frequency
0.2
0.4
0.6
0.8
FRA_1
SPA_4
ITA_3
ITA_6
GRE_3
GRE_6
GRE_9b

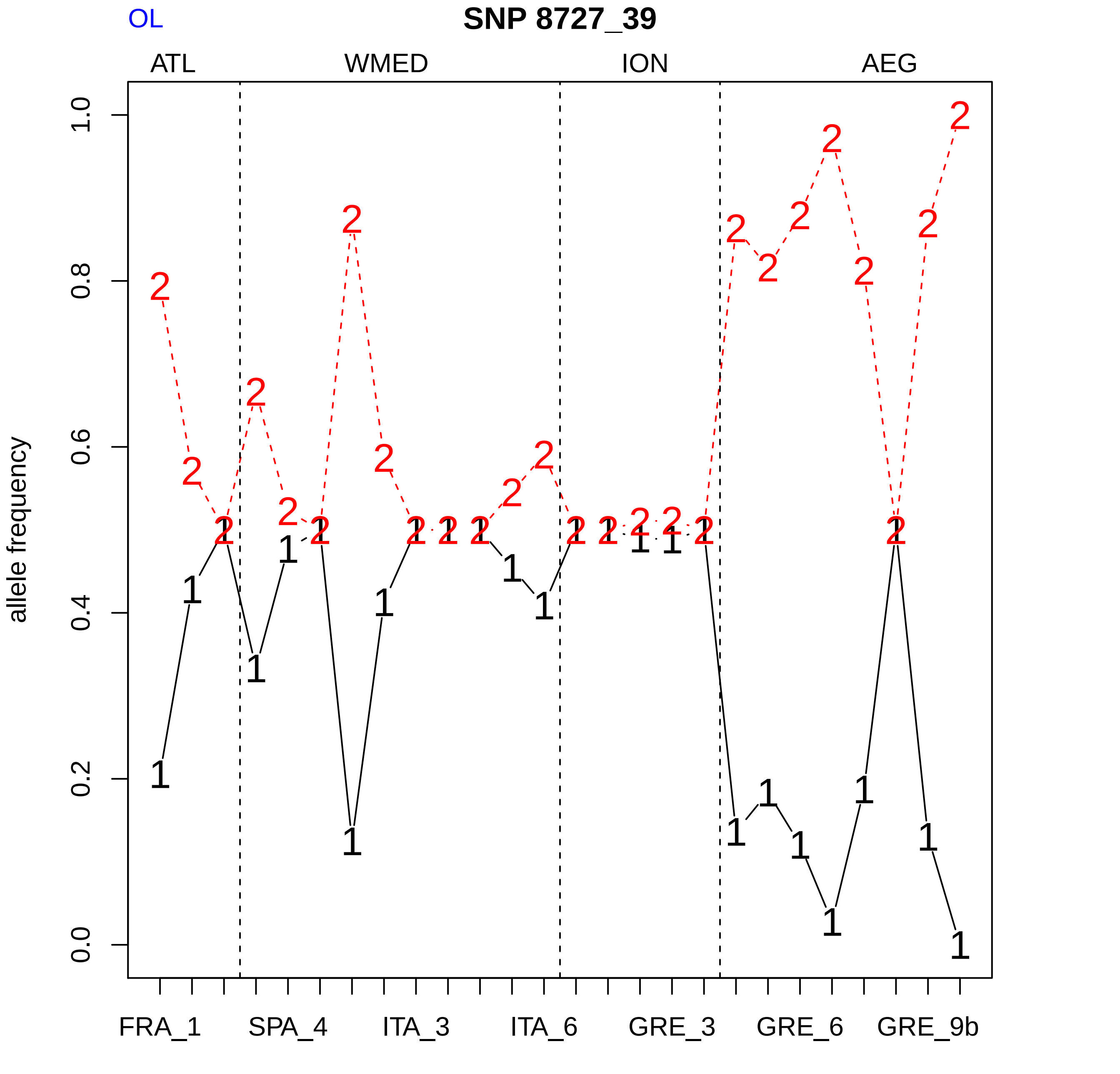
OL
SNP 8727_39
ATL
WMED
ION
AEG
allele frequency
0.0
0.2
0.4
0.6
0.8
1.0
FRA_1
SPA_4
ITA_3
ITA_6
GRE_3
GRE_6
GRE_9b

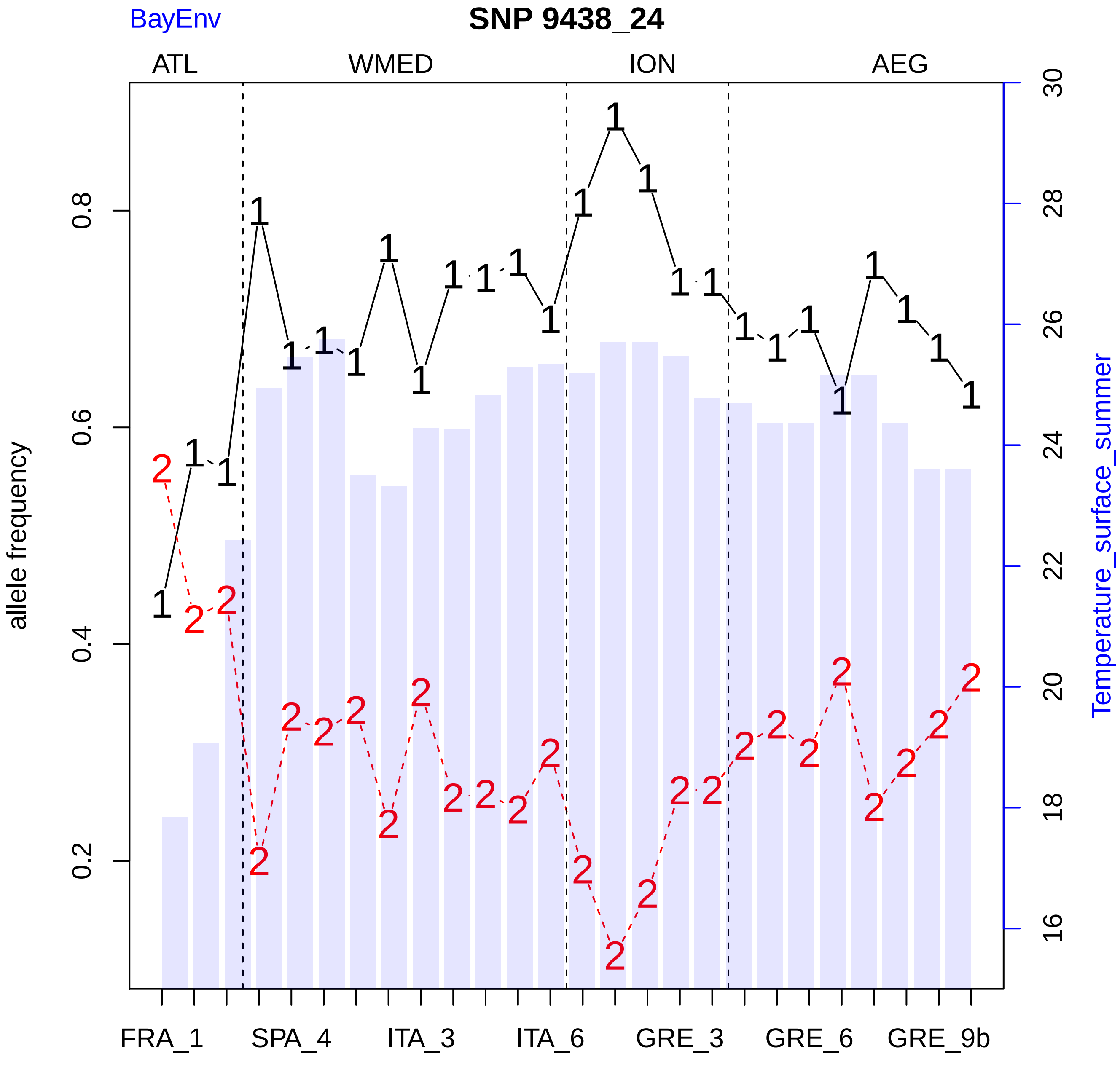
BayEnv
SNP 9438_24
ATL
WMED
ION
AEG
allele frequency
Temperature_surface_summer
0.2
0.4
0.6
0.8
16
18
20
22
24
26
28
30
FRA_1
SPA_4
ITA_3
ITA_6
GRE_3
GRE_6
GRE_9b

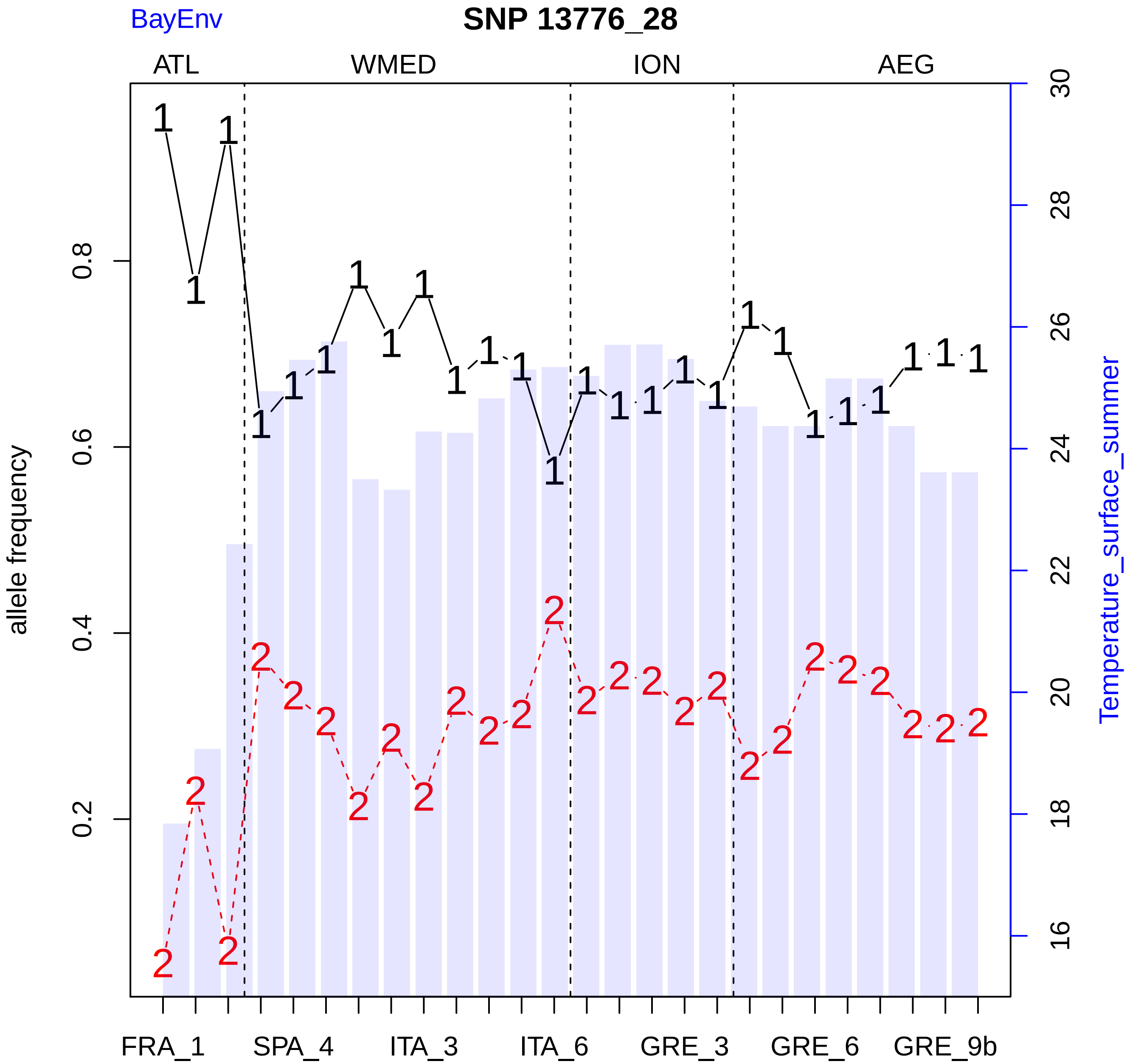
BayEnv
SNP 13776_28
ATL
WMED
ION
AEG
allele frequency
Temperature_surface_summer
FRA_1
SPA_4
ITA_3
ITA_6
GRE_3
GRE_6
GRE_9b

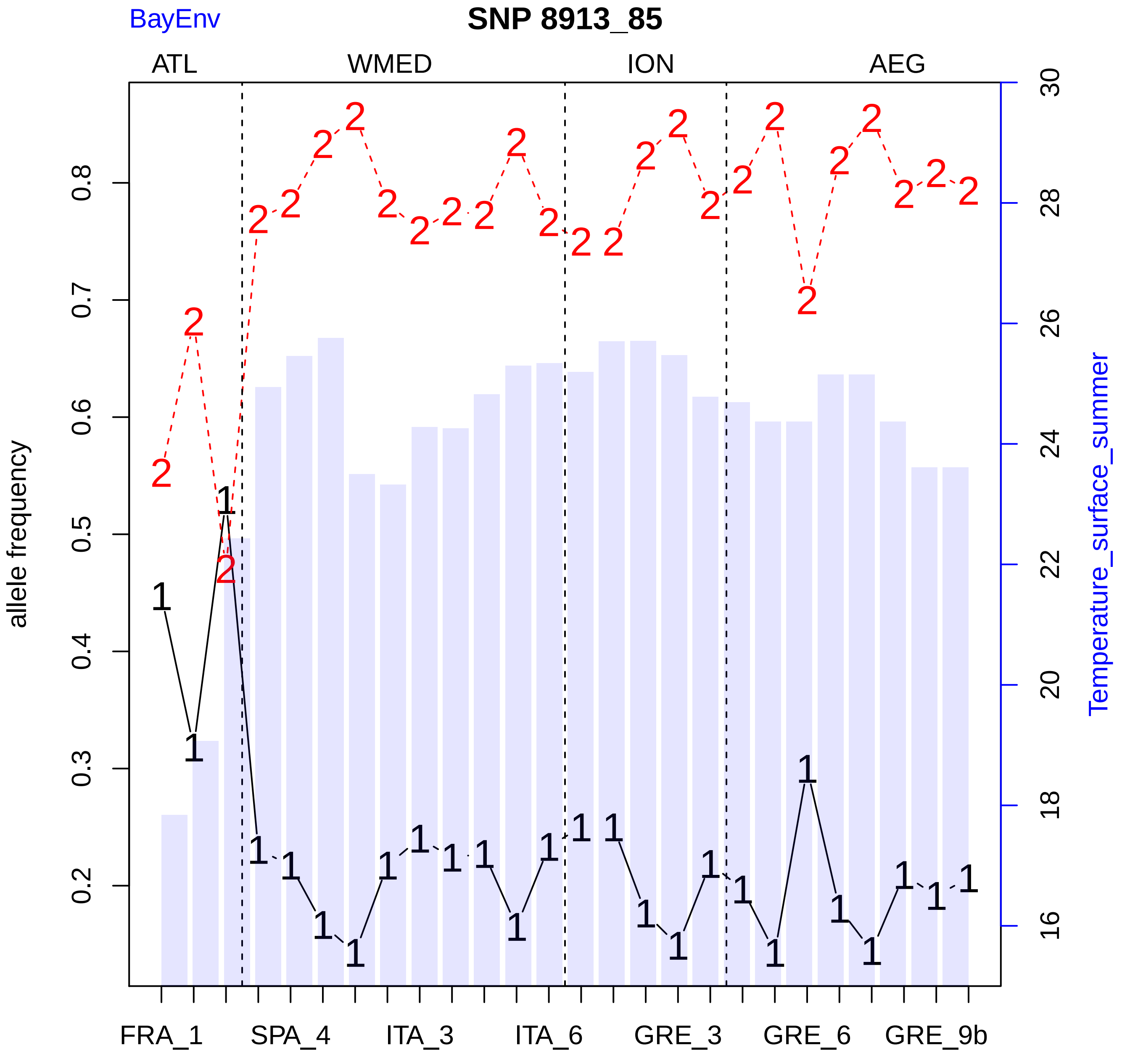

BayEnv
SNP 8913_85
ATL
WMED
ION
AEG
allele frequency
Temperature_surface_summer
0.2
0.3
0.4
0.5
0.6
0.7
0.8
16
18
20
22
24
26
28
30
FRA_1
SPA_4
ITA_3
ITA_6
GRE_3
GRE_6
GRE_9b

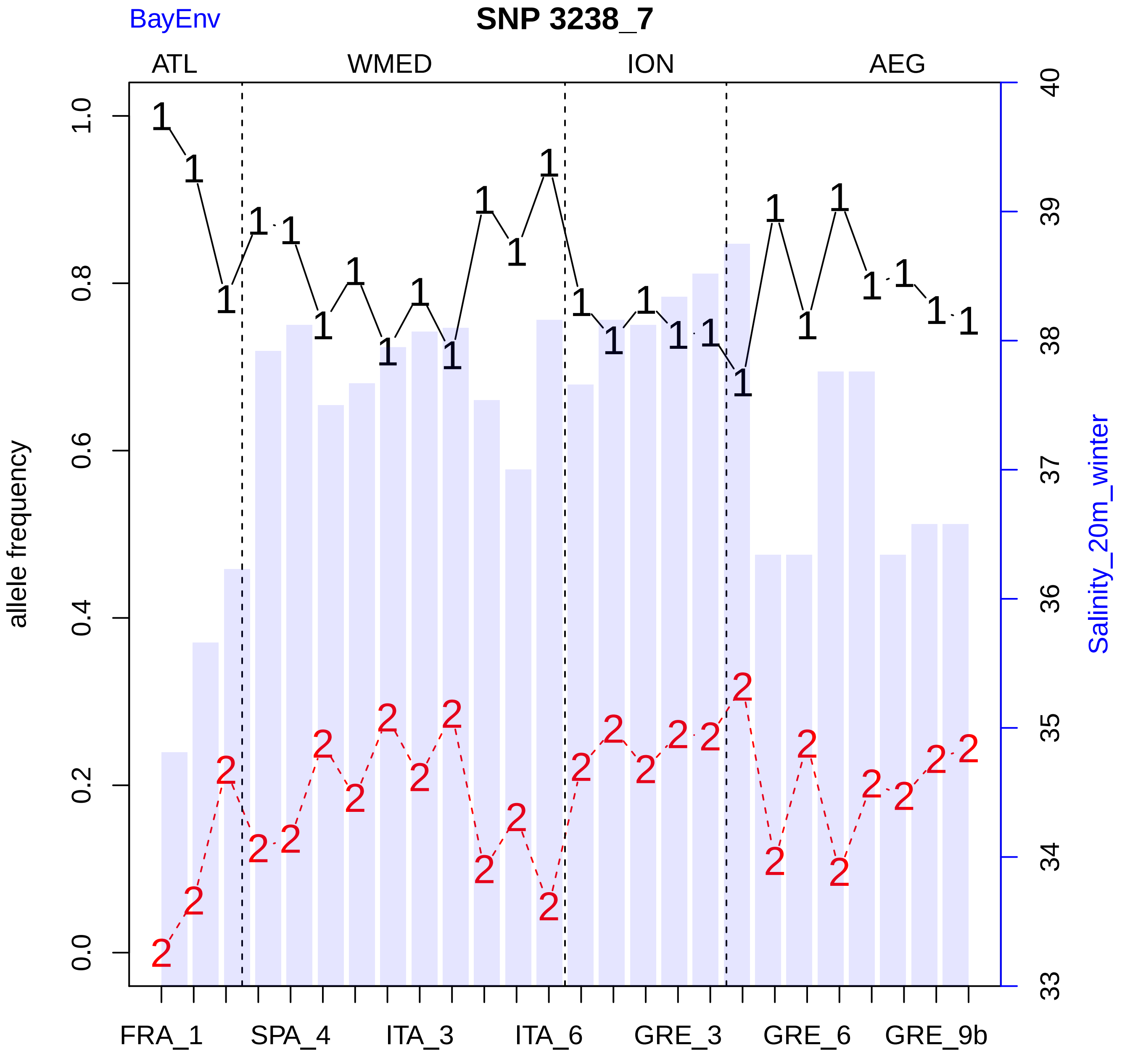

BayEnv
SNP 3238_7
ATL
WMED
ION
AEG
allele frequency
0.0
0.2
0.4
0.6
0.8
1.0
Salinity_20m_winter
33
34
35
36
37
38
39
40
FRA_1
SPA_4
ITA_3
ITA_6
GRE_3
GRE_6
GRE_9b

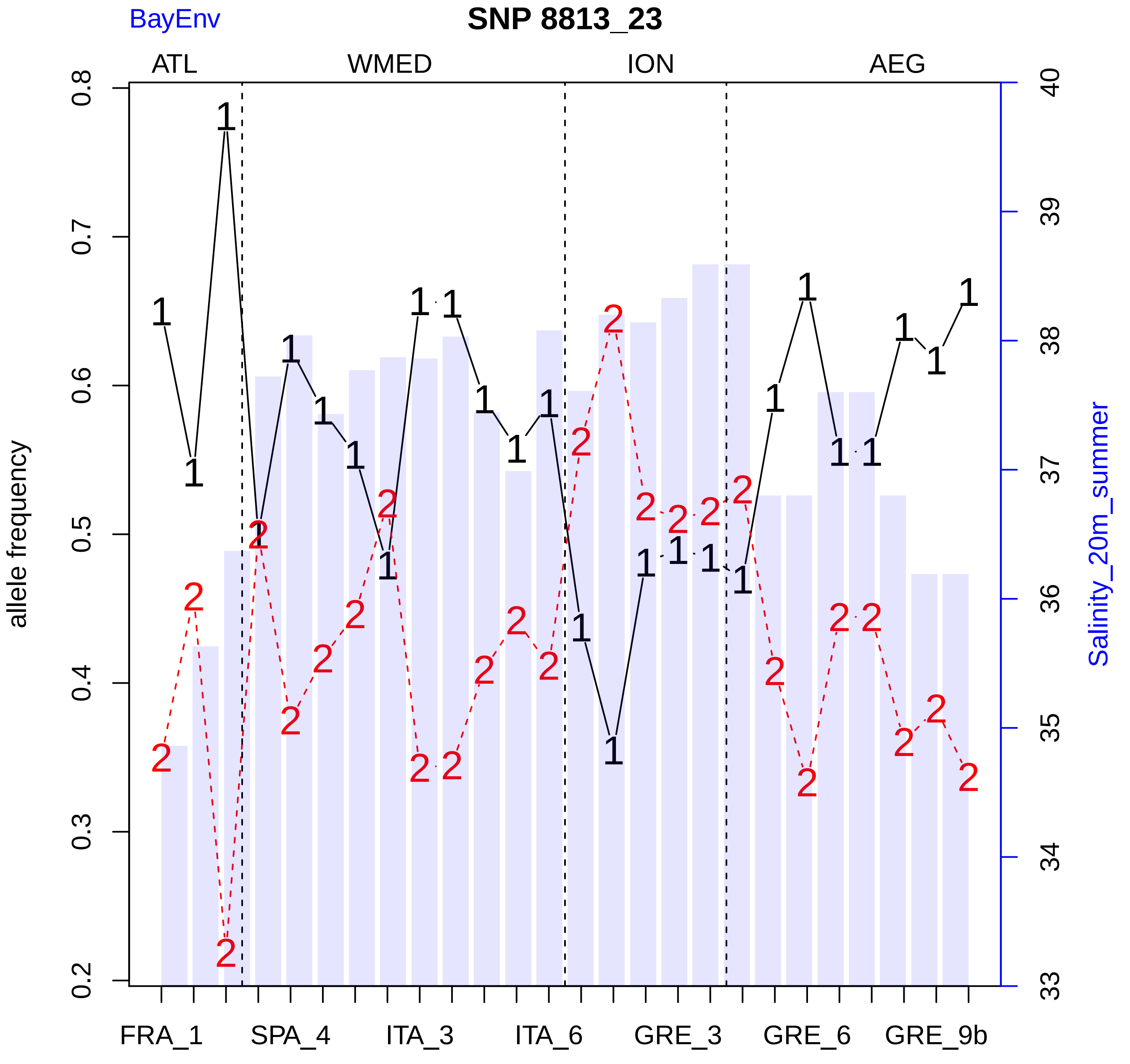

BayEnv
SNP 8813_23
ATL
WMED
ION
AEG
allele frequency
Salinity_20m_summer
0.2
0.3
0.4
0.5
0.6
0.7
0.8
33
34
35
36
37
38
39
40
FRA_1
SPA_4
ITA_3
ITA_6
GRE_3
GRE_6
GRE_9b

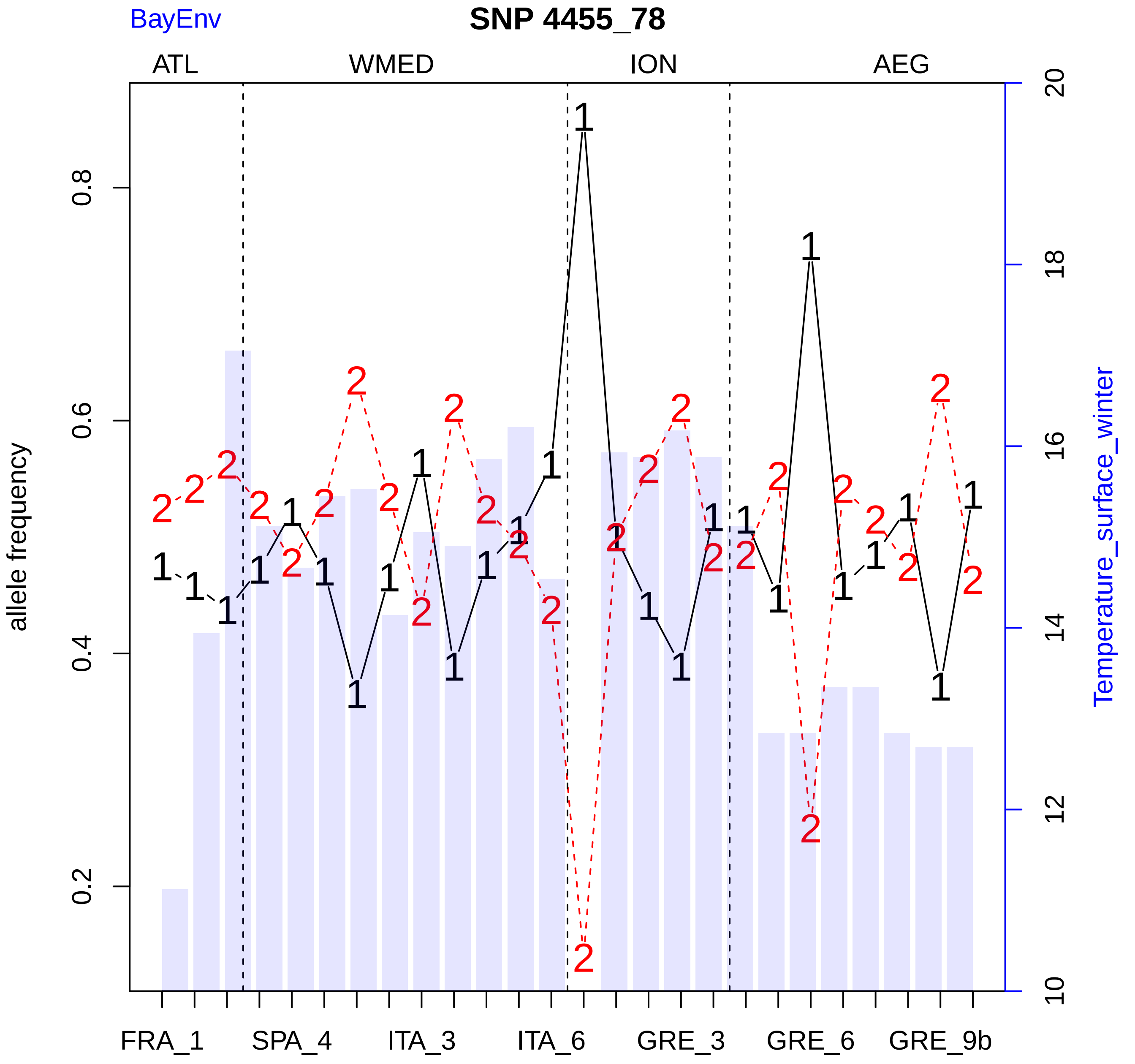

BayEnv
SNP 4455_78
ATL
WMED
ION
AEG
allele frequency
0.2
0.4
0.6
0.8
Temperature_surface_winter
10
12
14
16
18
20
FRA_1
SPA_4
ITA_3
ITA_6
GRE_3
GRE_6
GRE_9b

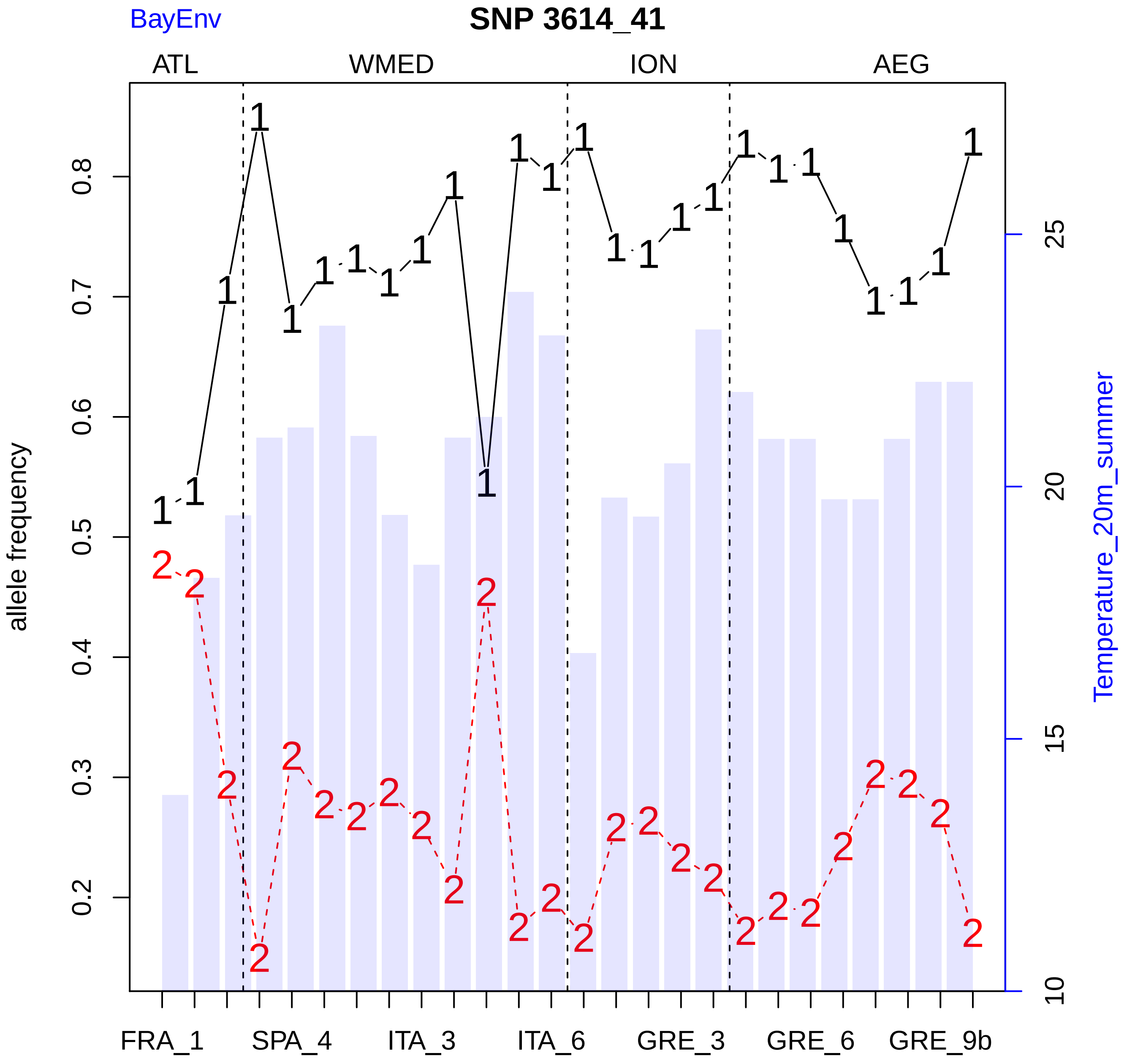

BayEnv
SNP 3614_41
ATL
WMED
ION
AEG
allele frequency
0.2
0.3
0.4
0.5
0.6
0.7
0.8
Temperature_20m_summer
10
15
20
25
FRA_1
SPA_4
ITA_3
ITA_6
GRE_3
GRE_6
GRE_9b

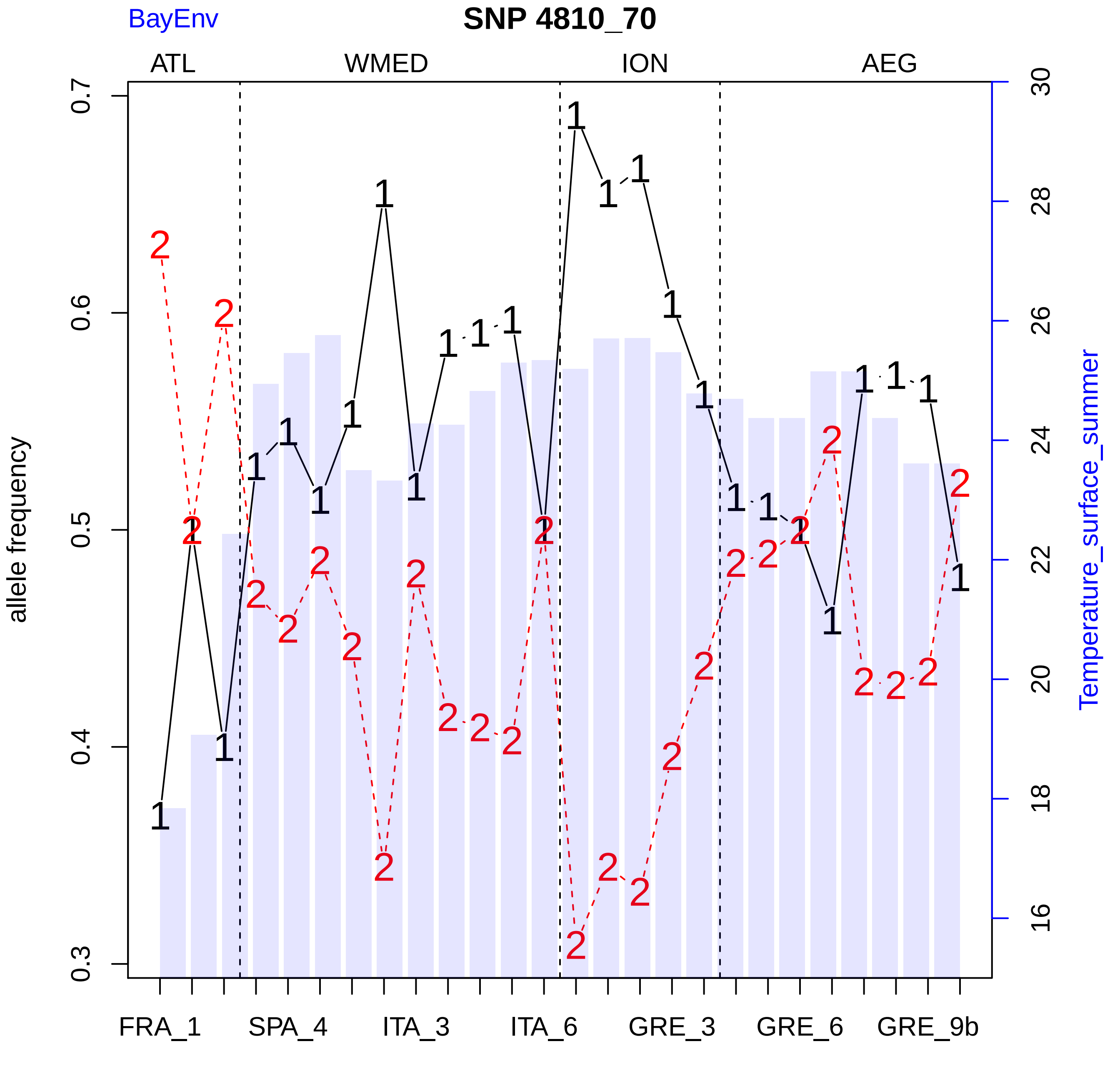

BayEnv
SNP 4810_70
ATL
WMED
ION
AEG
allele frequency
0.3
0.4
0.5
0.6
0.7
Temperature_surface_summer
16
18
20
22
24
26
28
30
FRA_1
SPA_4
ITA_3
ITA_6
GRE_3
GRE_6
GRE_9b

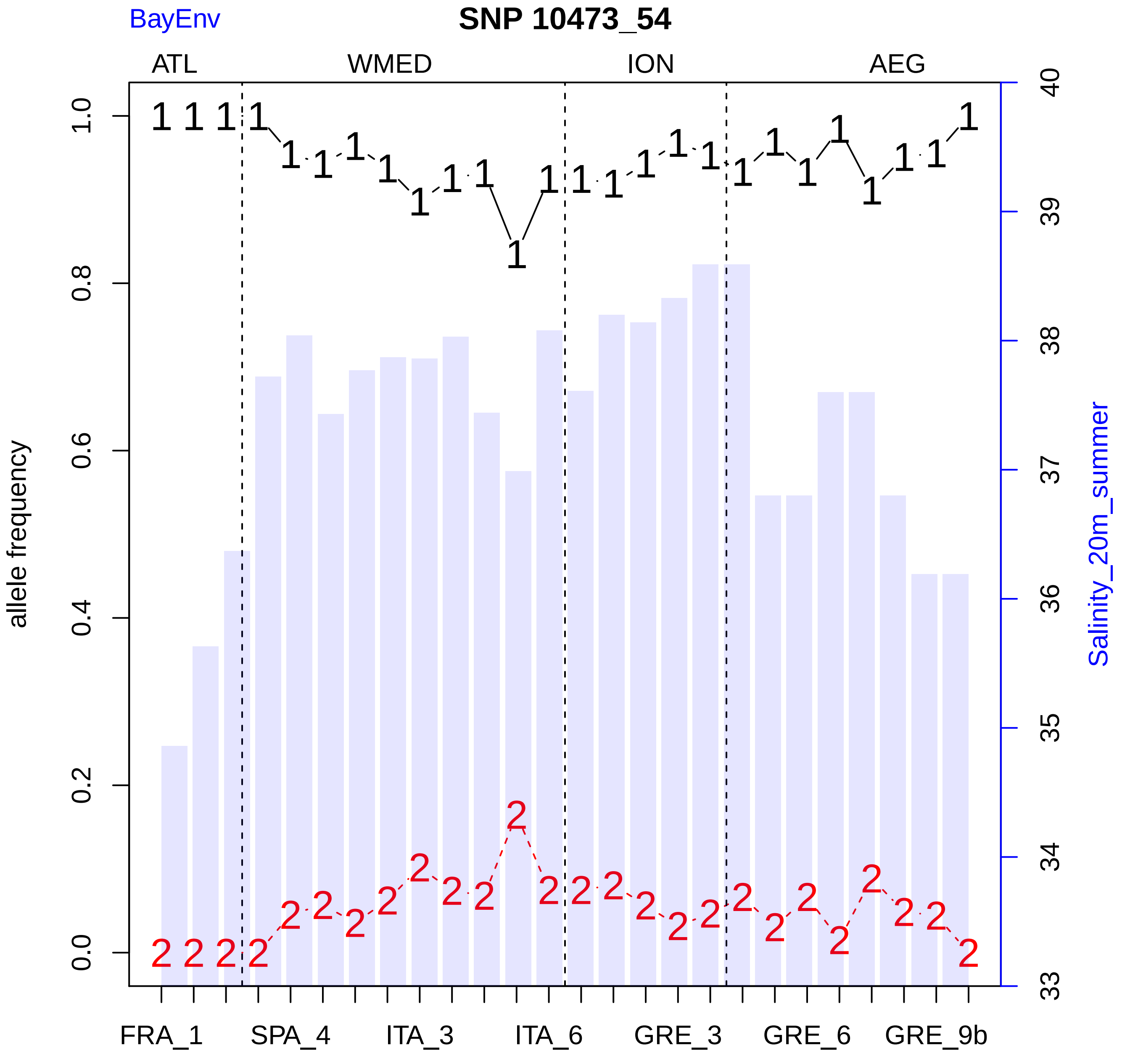

BayEnv
SNP 10473_54
ATL
WMED
ION
AEG
allele frequency
Salinity_20m_summer
0.0
0.2
0.4
0.6
0.8
1.0
33
34
35
36
37
38
39
40
FRA_1
SPA_4
ITA_3
ITA_6
GRE_3
GRE_6
GRE_9b

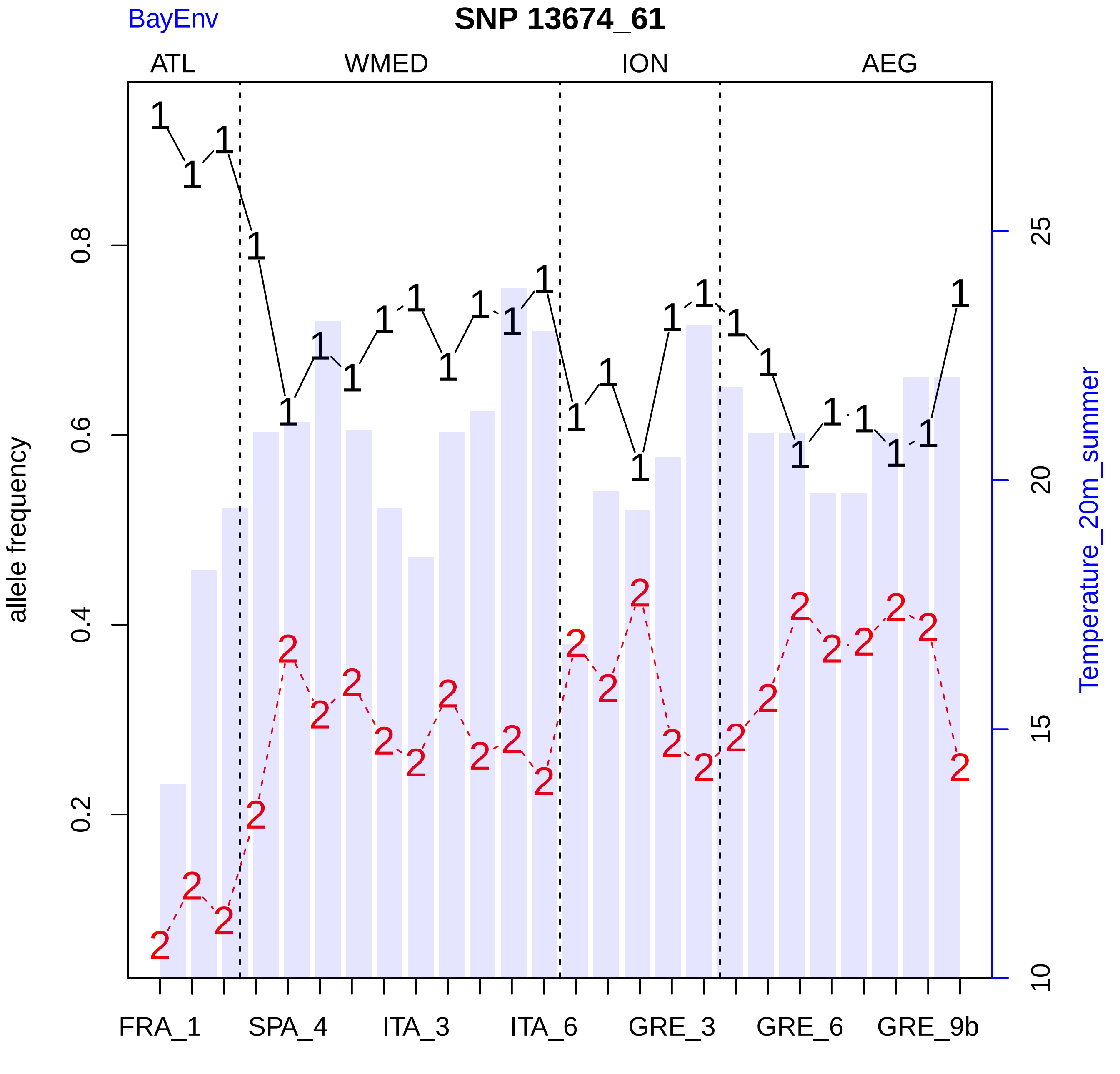
BayEnv
SNP 13674_61
ATL
WMED
ION
AEG
allele frequency
0.2
0.4
0.6
0.8
Temperature_20m_summer
10
15
20
25
FRA_1
SPA_4
ITA_3
ITA_6
GRE_3
GRE_6
GRE_9b

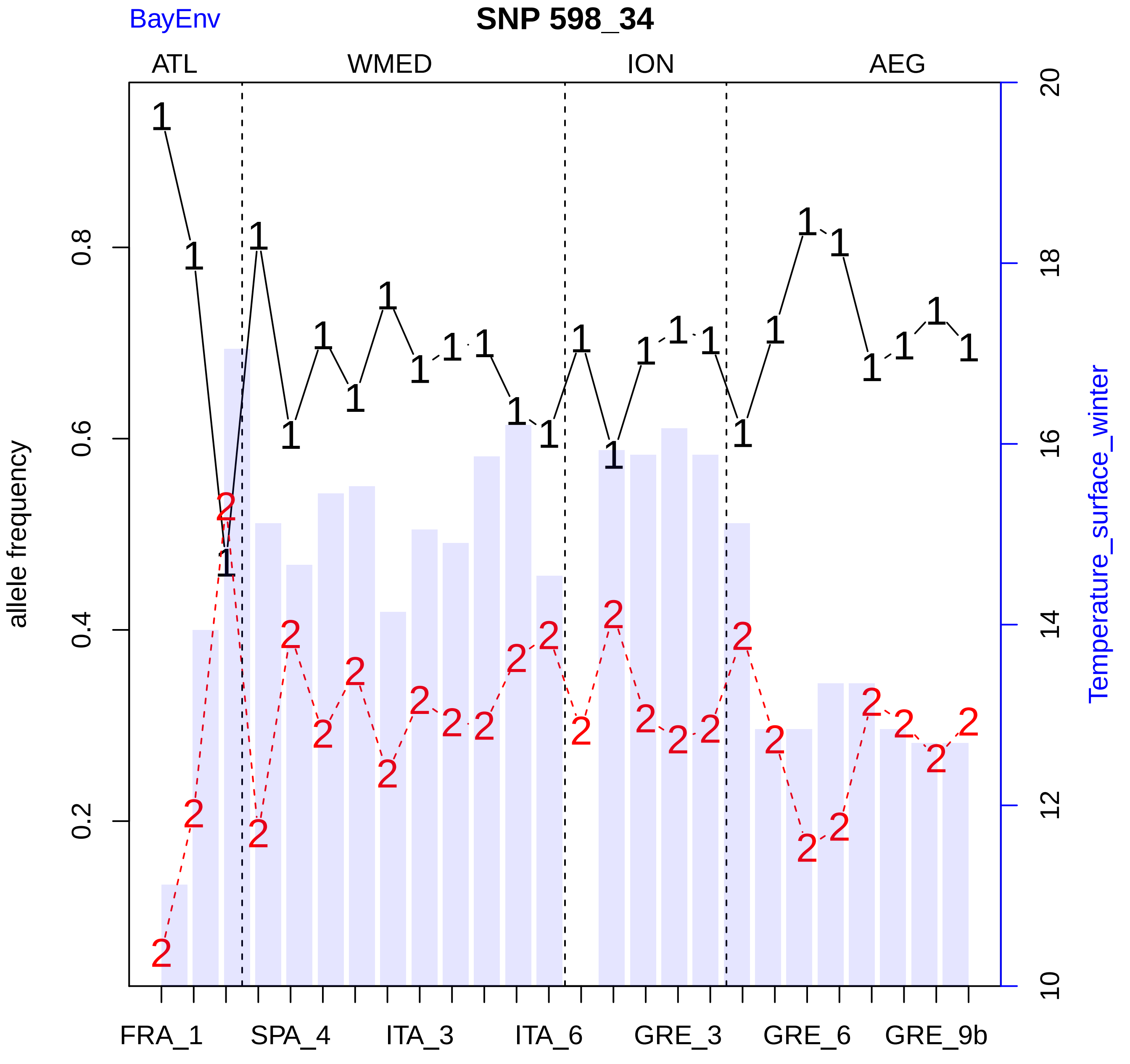

BayEnv
SNP 598_34
ATL
WMED
ION
AEG
allele frequency
0.2
0.4
0.6
0.8
Temperature_surface_winter
10
12
14
16
18
20
FRA_1
SPA_4
ITA_3
ITA_6
GRE_3
GRE_6
GRE_9b

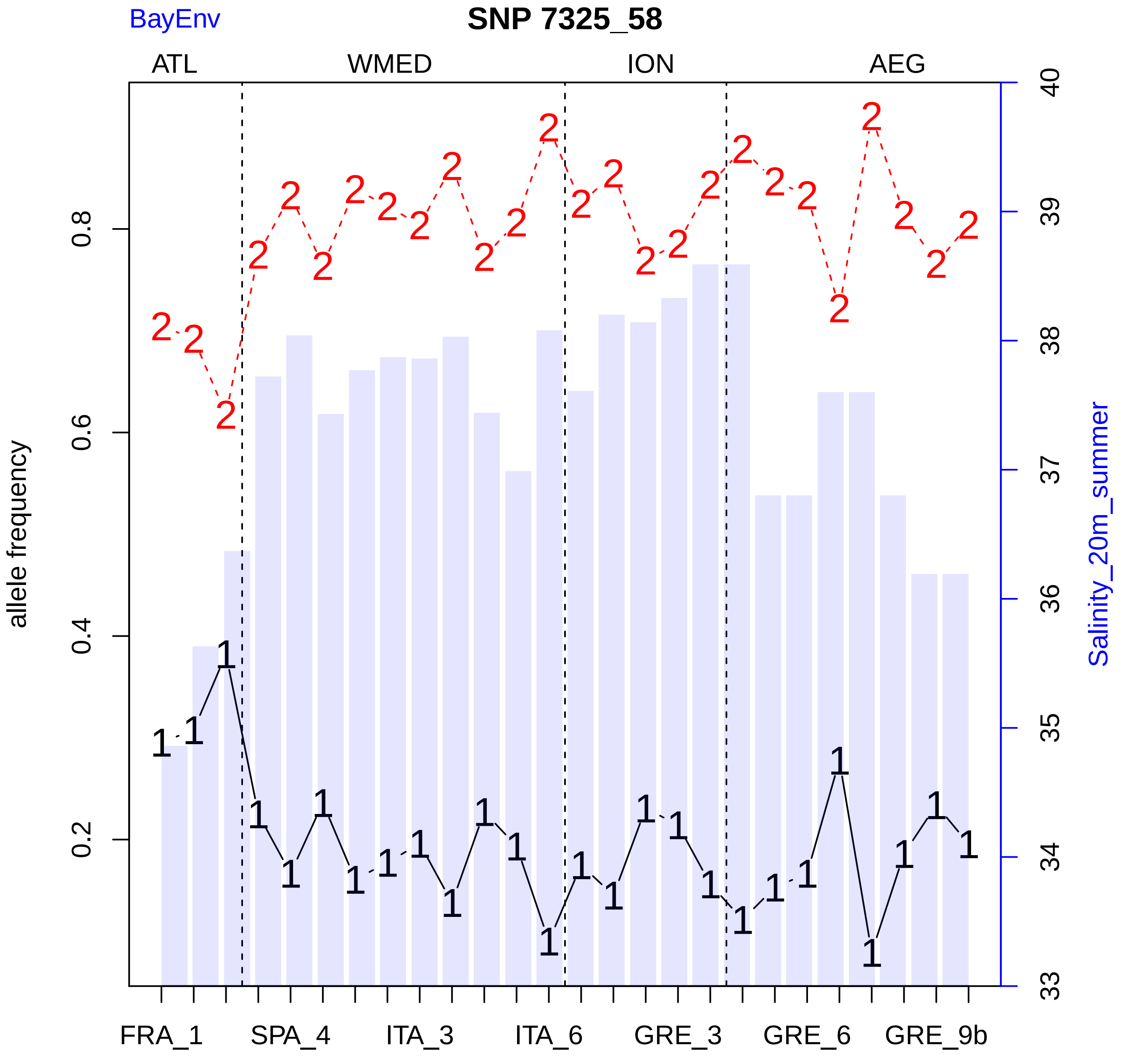
BayEnv
SNP 7325_58
ATL
WMED
ION
AEG
allele frequency
0.2
0.4
0.6
0.8
Salinity_20m_summer
33
34
35
36
37
38
39
40
FRA_1
SPA_4
ITA_3
ITA_6
GRE_3
GRE_6
GRE_9b

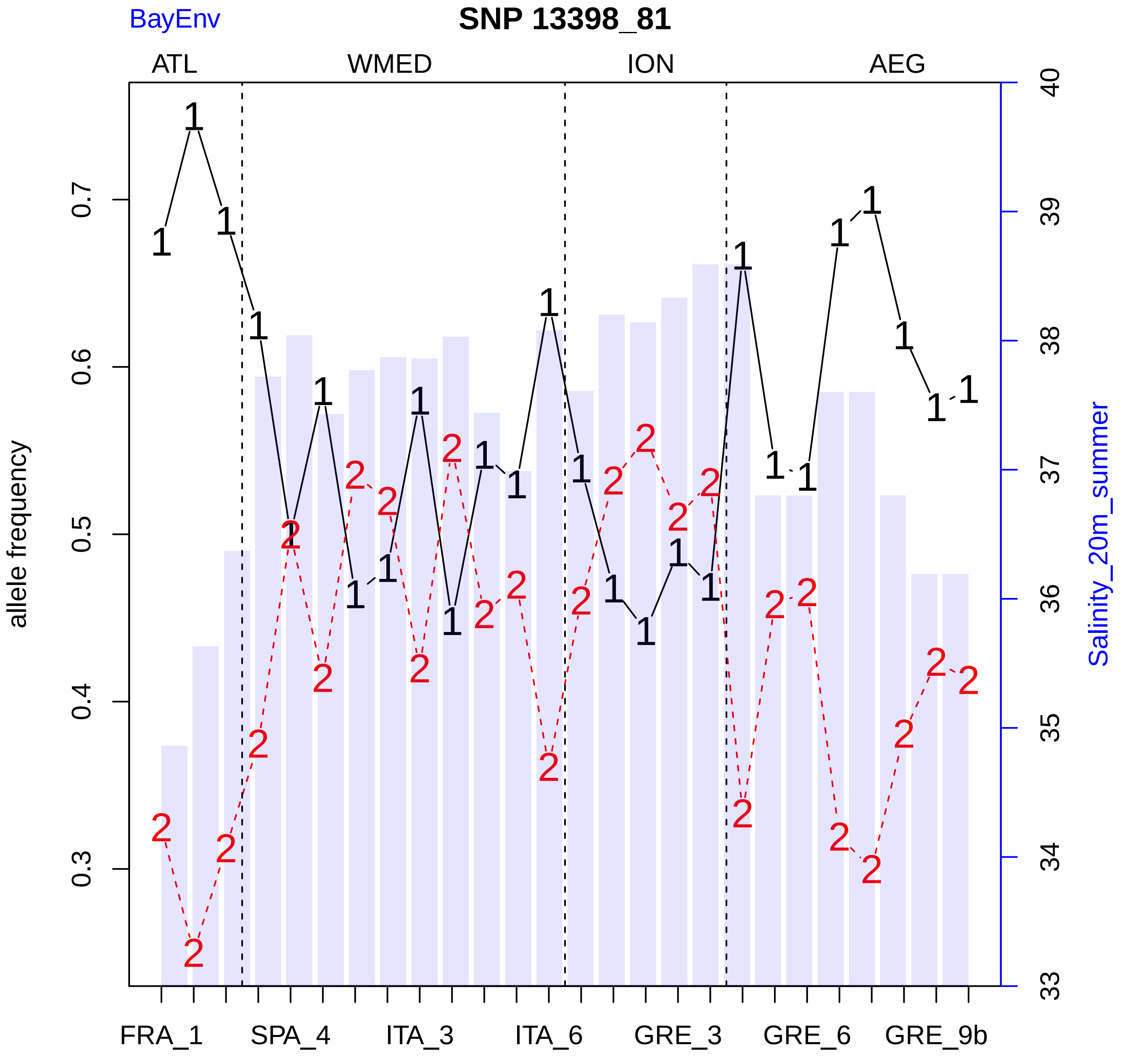

BayEnv
SNP 13398_81
ATL
WMED
ION
AEG
allele frequency
Salinity_20m_summer
0.3
0.4
0.5
0.6
0.7
33
34
35
36
37
38
39
40
FRA_1
SPA_4
ITA_3
ITA_6
GRE_3
GRE_6
GRE_9b

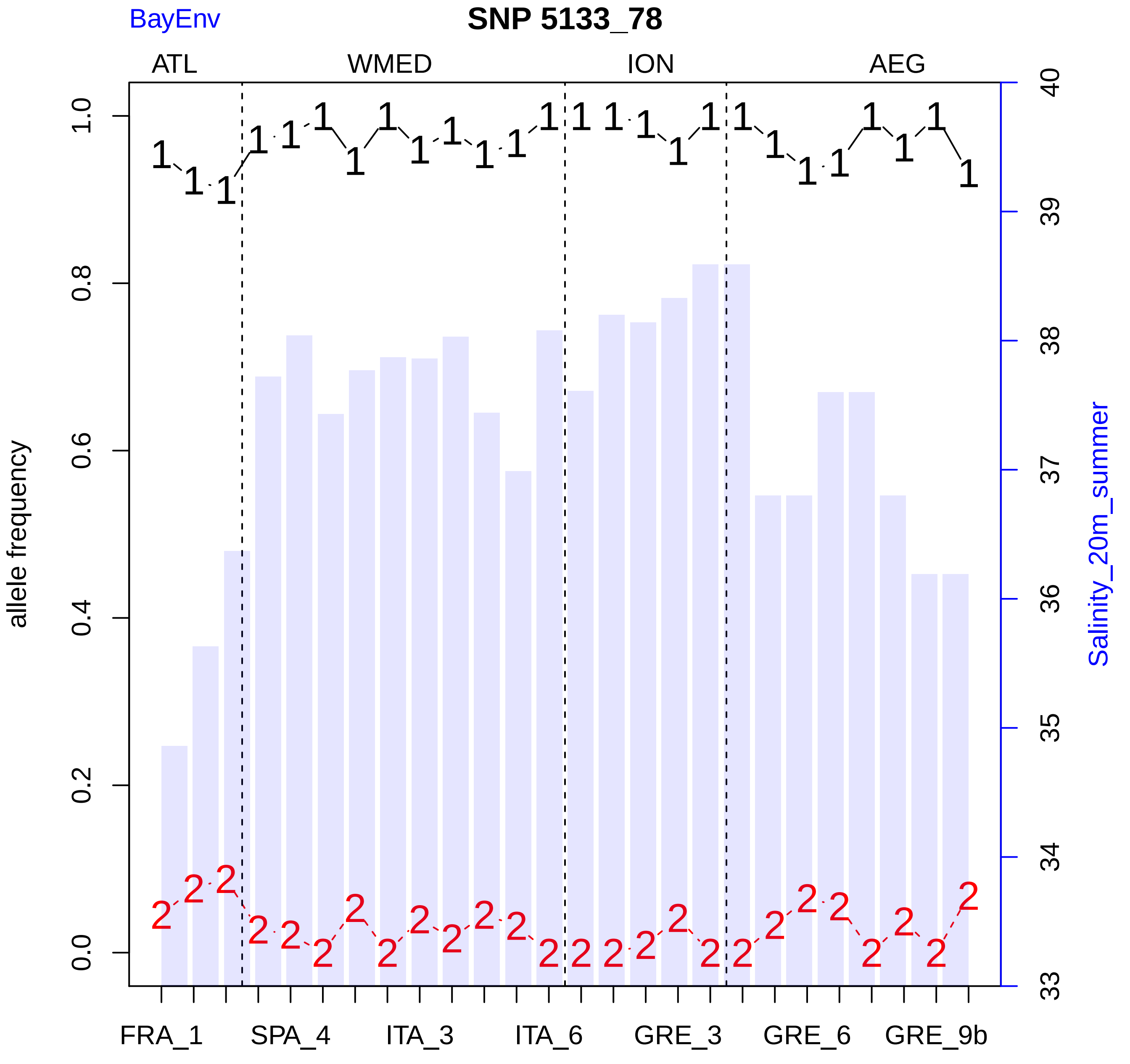
BayEnv
SNP 5133_78
ATL
WMED
ION
AEG
allele frequency
Salinity_20m_summer
0.0
0.2
0.4
0.6
0.8
1.0
33
34
35
36
37
38
39
40
FRA_1
SPA_4
ITA_3
ITA_6
GRE_3
GRE_6
GRE_9b

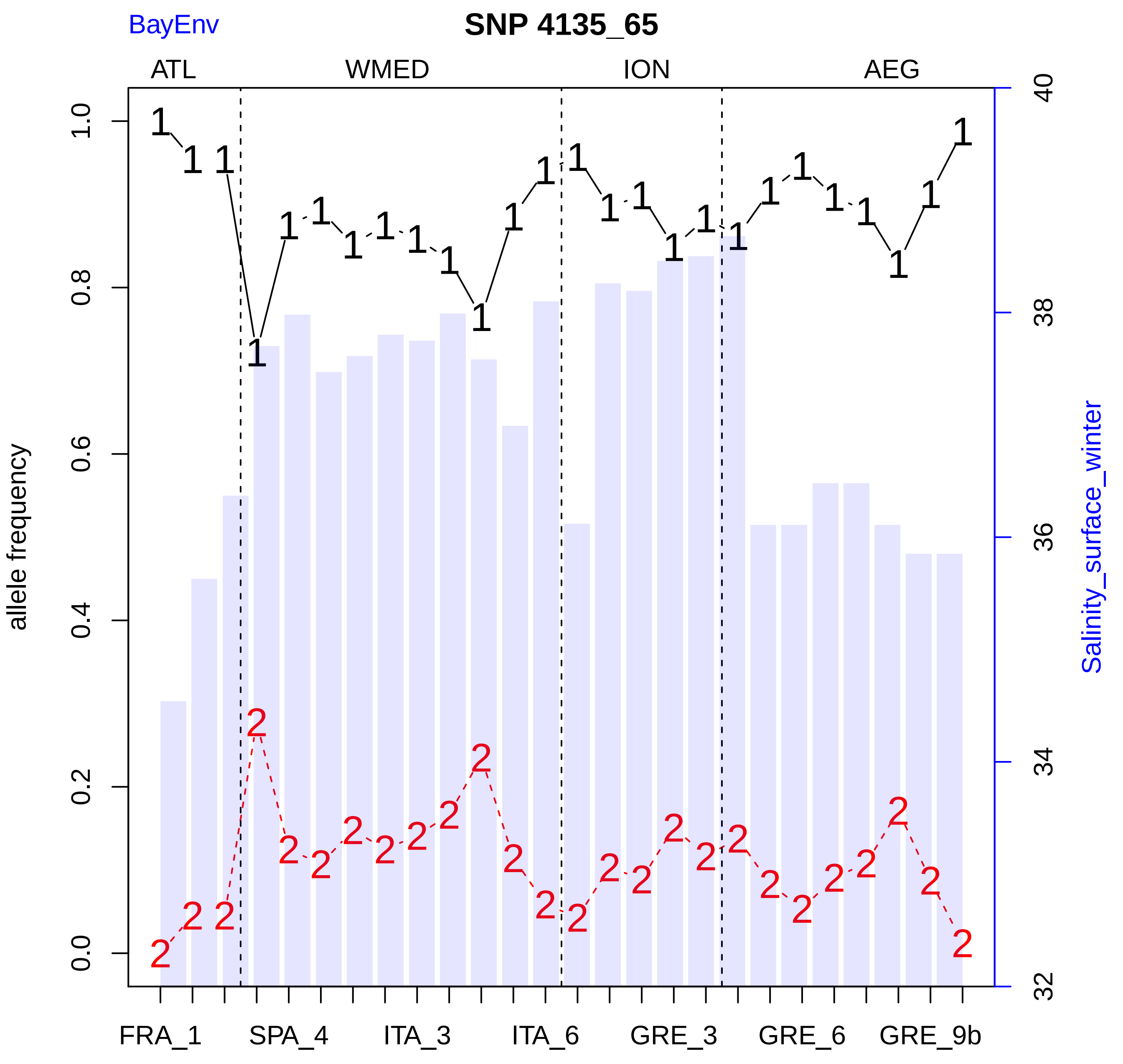

BayEnv
SNP 4135_65
ATL
WMED
ION
AEG
allele frequency
0.0
0.2
0.4
0.6
0.8
1.0
Salinity_surface_winter
32
34
36
38
40
FRA_1
SPA_4
ITA_3
ITA_6
GRE_3
GRE_6
GRE_9b

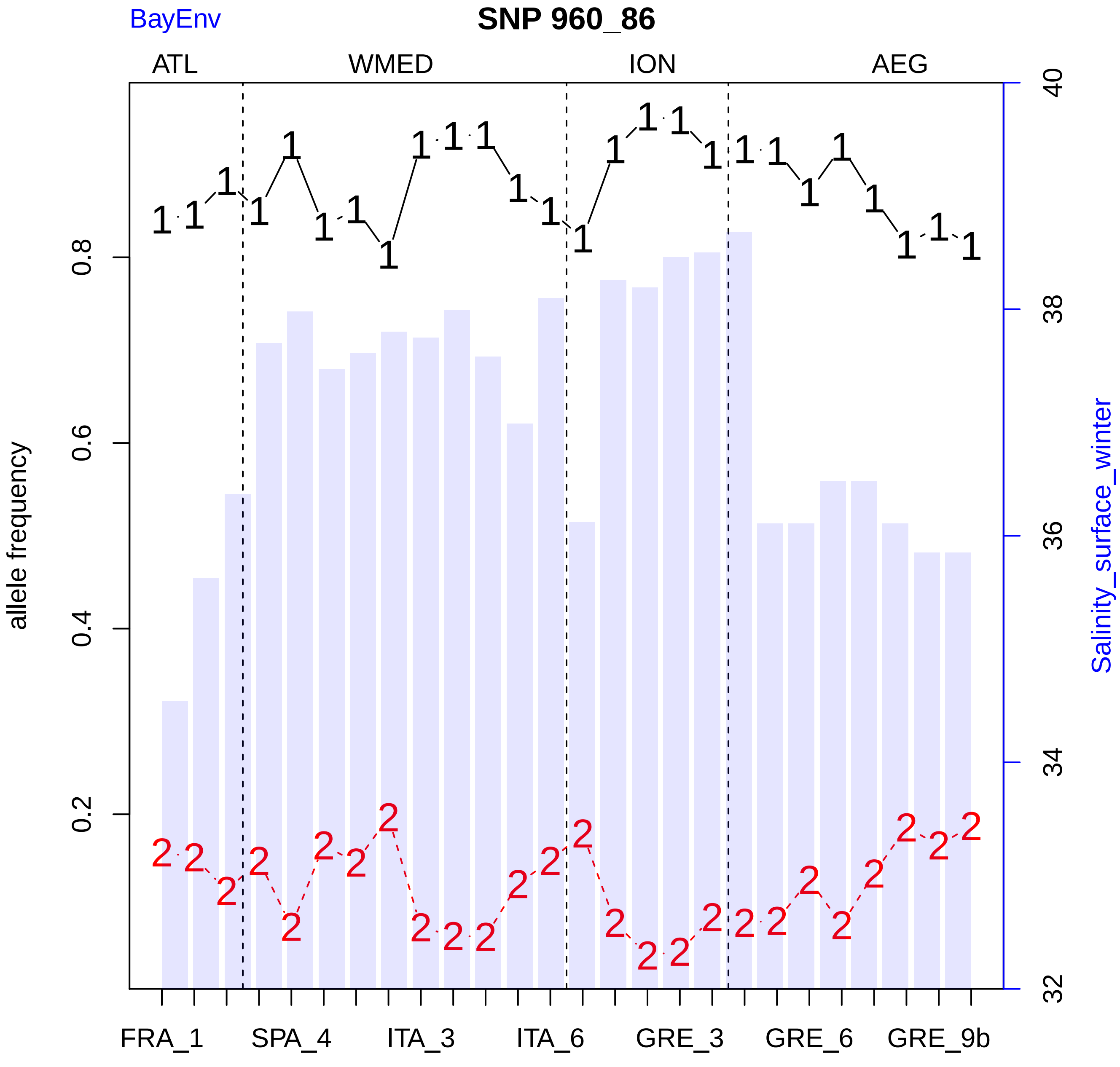

BayEnv
SNP 960_86
ATL
WMED
ION
AEG
allele frequency
0.2
0.4
0.6
0.8
Salinity_surface_winter
32
34
36
38
40
FRA_1
SPA_4
ITA_3
ITA_6
GRE_3
GRE_6
GRE_9b

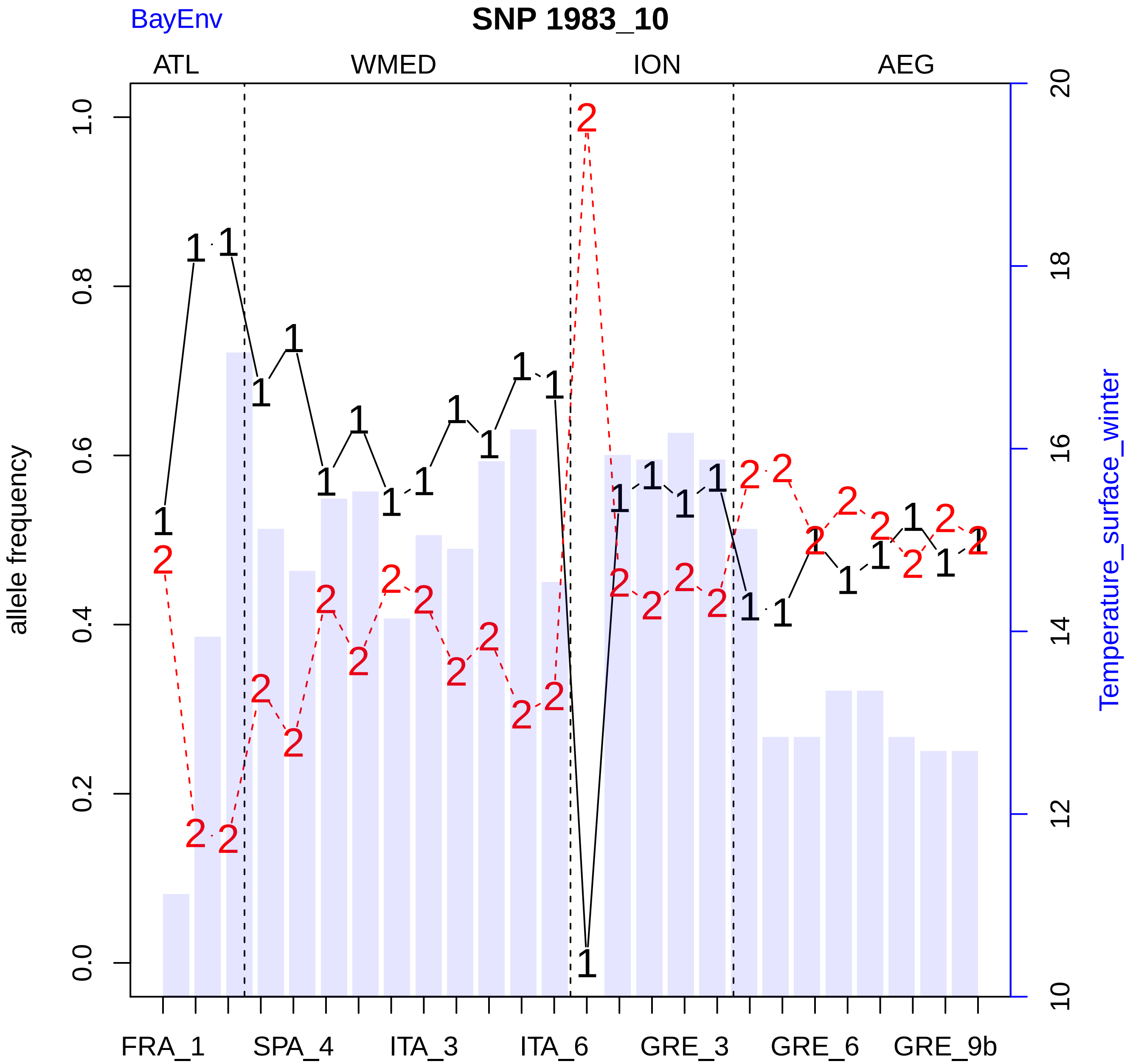
BayEnv
SNP 1983_10
ATL
WMED
ION
AEG
allele frequency
Temperature_surface_winter
0.0
0.2
0.4
0.6
0.8
1.0
10
12
14
16
18
20
FRA_1
SPA_4
ITA_3
ITA_6
GRE_3
GRE_6
GRE_9b

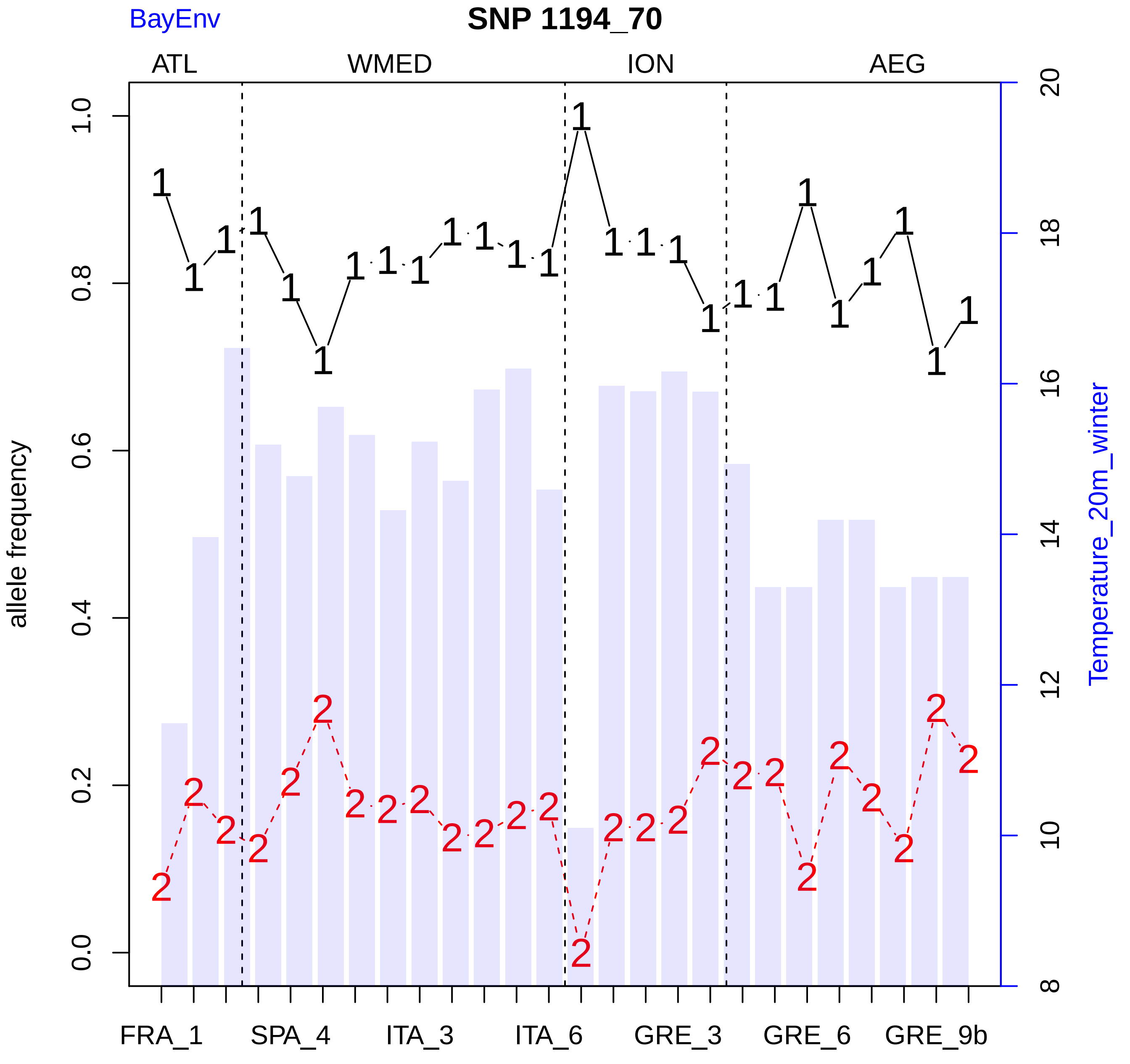

BayEnv
SNP 1194_70
ATL
WMED
ION
AEG
allele frequency
0.0
0.2
0.4
0.6
0.8
1.0
Temperature_20m_winter
8
10
12
14
16
18
20
FRA_1
SPA_4
ITA_3
ITA_6
GRE_3
GRE_6
GRE_9b
