# Supplementary material for: Genome-wide analysis clarifies the population genetic structure of wild gilthead sea bream (Sparus aurata)
Source: PLoS One. 2021 Jan 11;16(1):e0236230. doi: 10.1371/journal.pone.0236230 (PMC7799848; doi:10.1371/journal.pone.0236230)
Supplement: S1 File — (DOCX) [file pone.0236230.s010.docx]

**S1 File**

Detailed library preparation protocol

The original protocol of Peterson et al. (2012) involved processing each sample separately (i.e. restriction digestion, adapter ligation, fragment size selection, PCR amplification and purification, quantitation) prior to pooling into a single library for sequencing. A modified protocol (described in detail elsewhere; Palaikostas et al. 2014; Manousaki et al. 2016), which was more convenient for screening large numbers of individuals, was used for this project. The methodology allowed for pooling of samples after the adapter ligation step, which greatly reduced the number of manipulations required, ensured consistent size selection within libraries and reduced construction time to two to three working days. Library preparation began with basic qualitative and quantitative assessment of extracted DNA samples. DNA quality was evaluated by gel electrophoresis (0.8% agarose 0.5x TAE) and concentration was accurately measured by fluorimetry with each sample being finally diluted to 7 ng/µL in 5 mM Tris pH 8.5. For a library (144 samples), individual DNA samples (21 ng) were first simultaneously digested with *Sbf*I (recognition site CCTGCA'GG) and *Sph*I (recognition site GCATG'C) restriction enzymes. An adapter mix comprising individual-specific barcoded combinations of P1 (*Sbf*I-compatible) and P2 (*Sph*I-compatible) adapters (compatible with Illumina sequencing chemistry) were then added / ligated. Adapters were designed such that adapter– genomic DNA ligations did not reconstitute RE sites, residual RE activity limiting concatemerization of genomic fragments. Each adapter included an inline five- or seven-base barcode, allowing for post-sequencing identification of individuals (P1-P2 combinatorial barcoding). The ligation reactions were terminated by heat inactivation and all 144 samples combined in a single pool. Following column purification of the pooled sample, DNA fragments in the range of 320 bp to 590 bp were size selected by agarose gel electrophoresis, followed by gel-based column purification. The eluted size-selected DNA template was then PCR amplified (14 cycles, 400 uL volume), column purified down to a 50 uL volume and then subjected to a further clean-up using an equal volume of AMPure magnetic beads (Perkin-Elmer, UK) (used in sea bream and turbot), to maximize removal of small fragments (less than ca. 200 bp). The final library was eluted in c.20 µL10 mM Tris pH 8.5.

Libraries were sequenced on Illumina HiSeq 2500 sequencers with pair-end (PE) 100 base option to allow sequencing of both barcodes at the Genomics Core of the University of Leuven, Belgium.

**References**

Manousaki, T., Tsakogiannis, A., Taggart, J. B., Palaiokostas, C., Tsaparis, D., Lagnel, J., & Tsigenopoulos, C. S. (2016). Exploring a Nonmodel Teleost Genome Through RAD Sequencing—Linkage Mapping in Common Pandora, Pagellus erythrinus and Comparative Genomic Analysis. *G3: Genes| Genomes| Genetics*, *6*(3), 509-519.

Palaiokostas, C., Bekaert, M., Khan, M. G., Taggart, J. B., Gharbi, K., McAndrew, B. J., & Penman, D. J. (2015). A novel sex-determining QTL in Nile tilapia (Oreochromis niloticus). *BMC genomics*, *16*(1), 171.

Peterson, B. K., Weber, J. N., Kay, E. H., Fisher, H. S., & Hoekstra, H. E. (2012). Double digest RADseq: an inexpensive method for de novo SNP discovery and genotyping in model and non-model species. *PloS one*, *7*(5), e37135.
