# Supplementary material for: Genome-wide analysis clarifies the population genetic structure of wild gilthead sea bream (Sparus aurata)
Source: PLoS One. 2021 Jan 11;16(1):e0236230. doi: 10.1371/journal.pone.0236230 (PMC7799848; doi:10.1371/journal.pone.0236230)
Supplement: Supplementary file 7 [file pone.0236230.s005.odt]

T: temperature (° C); S: salinity (‰);

0: surface; 20: at 20 meters;

SUM: summer; WIN: winter;
