# Supplementary material for: Genome-wide analysis clarifies the population genetic structure of wild gilthead sea bream (Sparus aurata)
Source: PLoS One. 2021 Jan 11;16(1):e0236230. doi: 10.1371/journal.pone.0236230 (PMC7799848; doi:10.1371/journal.pone.0236230)
Supplement: Supplementary file 8 [file pone.0236230.s007.odt]

	FRA-1	SPA-1	SPA-2	SPA-3	SPA-4	SPA-5	ITA-1	ITA-2	ITA-3	ITA-4	ITA-5	TUN-1	ITA-6	ITA-7	GRE-1	GRE-2	GRE-3	GRE-4	GRE-5	GRE-6	GRE-7	GRE-8
FRA-1																						
SPA-1	0																					
SPA-2	0	0																				
SPA-3	0	0	0																			
SPA-4	0,0045*	0	0,0059*	0																		
SPA-5	0,0031	0	0,0059**	0	0,0008																	
ITA-1	0,0063**	0,0027	0,0057**	0	0	0,0018																
ITA-2	0,0062**	0,0011	0,0071**	0	0	0	0															
ITA-3	0,0053**	0,0007	0,0056**	0	0	0	0,0008	0														
ITA-4	0,0075**	0,0010	0,0082**	0	0	0	0,0012	0	0													
ITA-5	0,0061**	0	0,0065**	0	0,0017	0,0006	0,0014	0	0	0,0011												
TUN-1	0,0064**	0	0,0074**	0	0	0	0,002*	0	0,0013*	0,0018	0											
ITA-6	0,0103**	0,0046	0,007*	0,0044	0	0	0,0006	0,0025	0,0006	0	0,0001	0,0026*										
ITA-7	0,0087**	0,0029	0,0095**	0	0	0	0	0	0,0004	0,0012	0	0,0023**	0									
GRE-1	0,0145**	0,0039*	0,0135**	0	0,0015	0,0045**	0,0058**	0	0,0055**	0,0068**	0,0023	0,0044**	0,0011	0								
GRE-2	0,0146**	0,0055*	0,0134**	0	0,004**	0,004**	0,0034**	0	0,0031**	0,0026*	0,0029*	0,0046**	0,0017	0	0							
GRE-3	0,0127**	0,0041*	0,0127**	0	0,0016	0,0059**	0,0045**	0,0005	0,0047**	0,0045**	0,0032*	0,0051**	0,0009	0	0,0010	0,0012						
GRE-4	0,012**	0,0048*	0,0119**	0	0,0033*	0,0041**	0,0035*	0	0,005**	0,0054**	0,0031	0,0041**	0,0007	0	0,0022	0,0014	0,0029*					
GRE-5	0,0114**	0,0023	0,0117**	0	0,0027	0,0039**	0,0048**	0,0006	0,0039**	0,005**	0,0025	0,0044**	0,0021	0	0,0028*	0,0033**	0,0035**	0,007**				
GRE-6	0,0118**	0,0024	0,0141**	0	0,0018	0,0025*	0,0049**	0	0,0022*	0,0034*	0,0057**	0,0065**	0,0007	0,0021	0,0025*	0,0029*	0,0029*	0,0029*	0,0014			
GRE-7	0,0138**	0,0055*	0,0149**	0	0,0035*	0,0046**	0,006**	0,0026*	0,0042**	0,0064**	0,0034*	0,0052**	0,0029*	0,0015	0,0022*	0,0046**	0,0048**	0,007**	0	0,0031**		
GRE-8	0,0134**	0,0033	0,0111**	0	0,0022	0,0033**	0,0053**	0,0005	0,0029**	0,0042**	0,0022	0,0036**	0	0	0,0017	0,0041**	0,0028**	0,0065**	0,0016	0	0,0005	
GRE-9	0,0094**	0,0022	0,0081**	0	0,0020	0,0019	0,0054**	0	0,0019*	0,0032*	0,0032*	0,0034**	0,0007	0,0004	0,0021*	0,0041**	0,0035**	0,0051**	0,0023*	0,0001	0,0026**	0
