# Supplementary material for: Genome-wide analysis clarifies the population genetic structure of wild gilthead sea bream (Sparus aurata)
Source: PLoS One. 2021 Jan 11;16(1):e0236230. doi: 10.1371/journal.pone.0236230 (PMC7799848; doi:10.1371/journal.pone.0236230)
Supplement: Supplementary file 9 [file pone.0236230.s008.odt]

	FRA-1	SPA-1	SPA-2	SPA-3	SPA-4	SPA-5	ITA-1	ITA-2	ITA-3	ITA-4	ITA-5	TUN-1	ITA-6	ITA-7	GRE-1	GRE-2	GRE-3	GRE-4	GRE-5	GRE-6	GRE-7	GRE-8	GRE-9
FRA-1		-0.00605	-0.00163	-0.00226	0.00102	0.00116	0.00063	0.00365	0.00264	0.00378	0.0034	0.00058	0.00662	-0.04111	0.00876**	0.00905**	0.00599**	0.00431	0.00067	-0.00806	0	0.00468**	-0.0004
SPA-1	0.03105		-0.00293	-0.0026	-0.00803	-0.00611	-0.00363	0.00156	-0.00562	-0.00616	-0.00472	-0.00997	0.00544	-0.05571	-0.00235	0.00029	-0.00058	-0.00586	-0.00523	-0.01933	-0.00671	-0.00538	-0.0101
SPA-2	0.0436**	0		-0.00997	0.00391	0.00717**	0.00312	0.00404	0.00397	0.00361	0.00405	-0.00017	0.00337	-0.04038	0.01008**	0.01004**	0.00908**	0.0066	0.00618**	-0.00506	0.0016	0.00705**	2,0000
SPA-3	0.0538**	0.0065	0		-0.01924	-0.01943	-0.0178	-0.00194	-0.02038	-0.02201	-0.01563	-0.01872	0.00155	-0.06837	-0.01327	-0.01526	-0.01537	-0.01925	-0.02085	-0.03107	-0.01889	-0.02002	-0.0236
SPA-4	0.0964**	0.02672	0.0231	0		-0.00064	-0.00273	-0.00611	-0.00112	-0.00267	0.00021	-0.00488	-0.00668	-0.04371	0.00133	0.00289	0.00091	0.0015	-0.00005	-0.00974	-0.00588	0.00122	-0.00122
SPA-5	0.0483**	0	0	0	0		0.00016	-0.00596	-0.00062	0.00005	0.00079	-0.00702	-0.00468	-0.04566	0.0019	0.00214	0.00504**	0.00148	0.00142	-0.01257	-0.00462	0.00252	-0.00084
ITA-1	0.0639**	0.00853	0.01076	0	0	0		-0.00614	-0.00085	-0.00123	-0.00029	-0.00799	-0.00275	-0.05264	0.00203	0.00074	0.00158	-0.00084	-0.0006	-0.01122	-0.00371	0.00059	0.00021
ITA-2	0.0922**	0.0301	0.00239	0	0	0	0		-0.00651	-0.00556	-0.00311	-0.0089	0.00329	-0.05291	-0.00397	-0.00222	-0.00138	-0.00646	-0.00524	-0.01651	-0.00731	-0.00426	-0.00592
ITA-3	0.0632**	0.0221	0.02214	0	0.00099	0.00136	0	0		0.00038	-0.00067	-3,0000	-0.00368	-0.03792	0.00363**	0.00168	0.00265**	0.00255	0.00047	-0.00944	-0.00169	0.00324**	-0.00112
ITA-4	0.0667**	0	0.0182	0	0	0	0	0	0		0.00101	-0.00632	-0.0041	-0.04555	0.00388**	-0.00021	0.00192	0.00111	0.00047	-0.01249	-0.00309	0.00249	-0.00103
ITA-5	0.0825**	0.00803	0.0007	0	0	0	0	0	0	0		-0.00641	-0.00126	-0.05023	0.00077	0.00119	0.00215	0.00095	-0.00007	-0.01113	-0.00517	0.00034	0.00115
TUN-1	0.0486**	0.01455	0.00367	0	0.00229	0	0	0	0	0	0		-0.00546	-0.01767	-0.00187	-0.00264	-0.00124	-0.00151	-0.00459	-0.00098	0.00158	-0.00135	-0.00492
ITA-6	0.0737**	0.00099	0.00927	0	0	0	0	0.00259	0.00852	0	0	0		-0.05173	-0.0007	-0.00136	-0.00104	-0.00601	-0.00456	-0.01777	-0.00664	-0.00575	-0.00693
ITA-7	0.0751**	0	0.01226	0	0	0	0	0	0	0	0	0.0303**	0		-0.04214	-0.04385	-0.04133	-0.03767	-0.04503	-0.01153	-0.01816	-0.0353	-0.03978
GRE-1	0.1676**	0.0826**	0.0929**	0.0451**	0.0274**	0.0534**	0.03**	0.0375**	0.0338**	0.0351**	0.0287**	0.0545**	0.0512**	0		0.0004	0.00108	0.00005	0.00038	-0.01014	-0.00432	0.00217	0.00047
GRE-2	0.1751**	0.1**	0.12**	0.0577**	0.039**	0.0619**	0.0341**	0.0542**	0.0605**	0.0506**	0.0487**	0.0643**	0.038**	0	0.0318**		0.00071	0.00027	0.00094	-0.01068	-0.00371	0.00248**	0.00047
GRE-3	0.1283**	0.0684**	0.0845**	0.0315	0.01495	0.033**	0.01134	0.0352**	0.0195**	0.0166	0.0214	0.0324**	0.0277	0	0.01317	0.00461		0.00149	-0.00017	-0.01029	-0.00346	0.00235	0.00022
GRE-4	0.1618**	0.086**	0.0893**	0.0414**	0.0329	0.0426**	0.02024	0.0269	0.0333**	0.03**	0.0224	0.0321**	0.0268**	0	0.0461**	0.0258**	0.0182		0.00173	-0.00987	-0.00056	0.0035**	-0.00134
GRE-5	0.1207**	0.01399	0.0679**	0.041**	0.0648**	0.062**	0.0297**	0.0358**	0.0696**	0.0497**	0.041**	0.0574**	0	0	0.058**	0.06**	0.0572**	0.0712**		-0.01212	-0.00798	-0.00019	0.00156
GRE-6	0.1243**	0.01238	0.0408**	0	0.01524	0.00587	0	0	0.01255	0.00807	0	0.05**	6	0.01347	0.00588	0.0275**	0.01291	0.0253	0.033**		0.00019	-0.01008	-0.0107
GRE-7	0.1344**	0	0.0356**	0.00047	0.0368**	0.0258**	0.0227**	0	0.0403**	0.0218**	0.00204	0.0556**	0.00446	0.016**	0.0262**	0.0734**	0.0573**	0.0574**	0.0191**	0.00677		-0.00456	-0.00573
GRE-8	0.1175**	0.0253	0.0478**	0.00666	0.0364**	0.0324**	0.01088	0.00342	0.0227**	0.016**	0.00204	0.0227**	0.0053	0	0.0173	0.0366**	0.0171**	0.0203**	0.0225**	0	12,0000		-0.00214
GRE-9	0.0872**	0.01278	0.0495**	0.0271	0.0523**	0.045**	0.01235	0.0291**	0.0369**	0.0374**	0.0334**	0.0403**	0.00775	0	0.0472**	0.0714**	0.0463**	0.061**	0.0071	0.0305**	0.0282**	0.0205**	
